# Synthesis and Biological Evaluations of NO-Donating Oxa- and Aza-Pentacycloundecane Derivatives as Potential Neuroprotective Candidates

**DOI:** 10.3390/molecules23020308

**Published:** 2018-01-31

**Authors:** Rajan Sharma, Jacques Joubert, Sarel F. Malan

**Affiliations:** Pharmaceutical Chemistry, School of Pharmacy, University of the Western Cape, Private Bag X17, Bellville 7535, South Africa; rsharma@uwc.ac.za (R.S.); jjoubert@uwc.ac.za (J.J.)

**Keywords:** NO-donating, *S*-nitrosylation, neuroprotection, polycyclic cage, calcium influx

## Abstract

In order to utilize the neuroprotective properties of polycyclic cage compounds, and explore the NO-donating ability of nitrophenyl groups, an array of compounds was synthesized where the different nitrophenyl groups were appended on oxa and aza-bridged cage derivatives. Biological evaluations of the compounds were done for cytotoxicity, neuroprotective abilities, the inhibition of *N*-methyl-d-aspartate (NMDA)-mediated Ca^2+^ influx, the inhibition of voltage-mediated Ca^2+^ influx, and *S*-nitrosylation abilities. All of the compounds showed low toxicity. With a few exceptions, most of the compounds displayed good neuroprotection and showed inhibitory activity for NMDA-mediated and voltage-gated calcium influx, ranging from high (>70%) to low (20–39%) inhibition. In the *S*-nitrosylation assay, the compounds with the nitro moiety as the NO-donating group exhibited low to good nitrosylation potency compared to the positive controls. From the biological evaluation of the tested compounds, it was not possible to obtain a simple correlation that could explain the results across all of the biological study domains. This can be ascribed to the independent processes evaluated in the different assays, which reiterate that neuroprotection is a result of multifactorial biochemical mechanisms and interactions. However, these results signify the important aspects of the pentacylcoundecylamine neuroprotectants across different biological study realms.

## 1. Introduction

Many of the most devastating neurological disorders are neurodegenerative. The prevalence of neurodegenerative disorders is increasing worldwide with the aging population [1]. Neurodegeneration is a general term for the selective and progressive loss of the structure and function of specific populations of neurons, and is observed in disorders such as Parkinson’s disease (PD), Alzheimer’s disease (AD), Huntington’s disease (HD), Amyotrophic lateral sclerosis (ALS or Lou Gehrig’s disease), and glaucoma. As per the World Health Organization (WHO) report on neurological disorders, the global burden of disease estimates and projection for neurodegenerative diseases such as Alzheimer’s, dementia, Parkinson’s disease, and multiple sclerosis rank second after cerebrovascular disease in terms of disability-adjusted life years [2].

Current research implicates excitotoxicity in a variety of neuropathological conditions, suggesting that neurodegenerative diseases with distinct aetiologies may have excitotoxicity as a common pathway. This process takes place following the over-activation of receptors for excitatory neurotransmitters such as the *N*-methyl-d-aspartate (NMDA) and α-amino-3-hydroxy-5-methyl-4-isoxazolepropionate (AMPA) receptors. Excitotoxins such as NMDA and kainic acid, as well as high levels of glutamate, cause excitotoxicity. The excessive activation of glutamate receptors such as the NMDA receptor leads to a number of damaging consequences, including the disturbance of calcium homeostasis, formation of free radicals, activation of mitochondrial permeability transition, and secondary excitotoxicity [3].

Neurodegenerative diseases may be caused by different mechanisms, but they share a final common pathway to neuronal injury in the form of the overstimulation of glutamate receptors, especially of the NMDA subtype [4]. The physiological role of the NMDA receptor is related to synaptic plasticity, which is mediated by the entry of calcium ions through the NMDA receptor-associated channel. However, the overactivation of NMDA receptors causes an excessive calcium ion influx, which triggers a series of cytoplasmic and nuclear processes leading to neuronal cell death. Hence, NMDA receptor antagonists/modulators could have potential therapeutic benefits. Apart from NMDA receptors, calcium influx through voltage-gated calcium channels (VGCCs) is also implicated in excitotoxicity. Due to the relevance of NMDA receptors and excitotoxic processes, research to antagonize or desensitize NMDA receptors as a therapeutic tool has been extremely dynamic [5]. There is also extensive data to show the advantage of drugs acting on VGCCs in neurodegenerative diseases [6], thus suggesting that VGCCs could be targeted for achieving neuroprotection.

The NMDA receptor has an *S*-nitrosylation site as a modulatory site that is located towards the N-terminus, and hence the extracellular region, of the receptor. NMDA receptor activity can be modulated by *S*-nitrosylation, in which the transfer of the NO group to a cysteine sulfhydryl takes place to form a RS–NO. This *S*-nitrosylation results in a decrease in the channel opening, and thus the downregulation of receptor/channel activity, avoidance of excessive Ca^2+^ entry, and neuroprotection [7]. This modulation of NMDA receptor activity can be utilized in the development of neuroprotective drugs.

**Figure 1:**
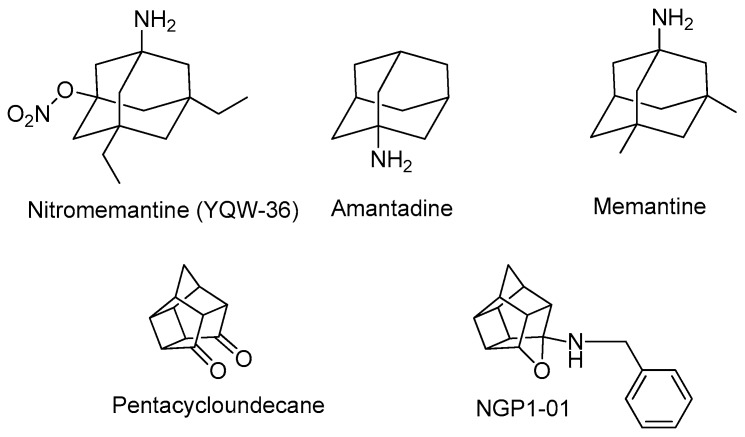
Polycyclic cage structures.

Polycyclic cage compounds such as amantadine, nitromemantine, and pentacycloundecane (Figure 1) have a relatively rigid conformation that can minimize the loss of conformational entropy on binding to a protein/receptor/substrate, and also can be used to improve and modify the pharmacokinetic and pharmacodynamic properties of pharmaceutically important chemical moieties [8]. Polycyclic compounds thus provide a convenient platform for further chemical transformations as side chain attachments and can improve the lipophilicity of the drug [9].

Various derivatives of different polycyclic cage compounds have been used for the design and synthesis of potential drugs against neurodegenerative diseases (Alzheimer’s disease and Parkinson’s disease) [8,9,10] and infectious diseases (malaria and dengue) [11,12].

Polycyclic structures such as adamantane derivatives and memantine block excessive NMDA receptor and VGCC activity without disturbing normal function, and have been the centre of active research in the field of neuroprotection for many years [8,13,14]. Following a similar therapeutic strategy, current research used polycyclic structures to inhibit excitotoxicity and provide a molecular platform to carry nitric oxide-donating moieties across the blood–brain barrier.

The strategy here was thus to synthesize compounds where NO-donating moieties are linked to polycyclic cage compounds with the hypothesis that the polycyclic cage structures bearing NO-donating groups can act in a synergistic way to yield potential neuroprotective candidates when the neuroprotective properties of polycyclic cage scaffolds and *S*-nitrosylation properties of NO-donating groups are combined.

**Figure 2:**
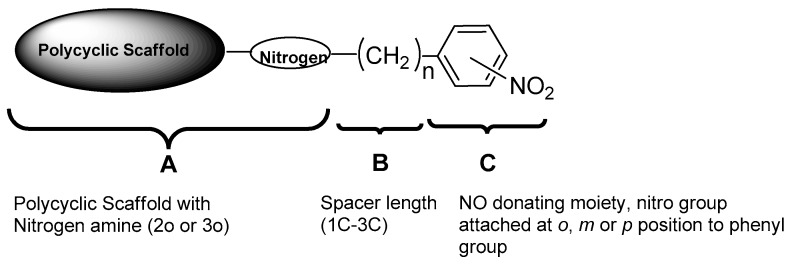
Pictorial representation of the general structure of synthesized compounds.

The general structure for the series of compounds synthesized is presented in Figure 2. The structure of all of the compounds in this series can be divided into three parts as A, B, and C. Part ‘A’ represents the polycyclic scaffold with a nitrogen present either as a 2° or 3° amine, to which a carbon spacer length of 1C to 3C is attached, as represented by part ‘B’. Part ‘C’ embodies the NO-donating moiety, which is a phenyl group with a nitro group attached at the *ortho*, *meta*, or *para* position. Among the polycyclic scaffolds, oxa and aza-pentacycloundecane derivatives were selected for synthesizing the compounds.

A series of compounds was proposed with various structural combinations of parts A, B, and C in order to investigate structure–activity relationships and the different structural aspects in these molecules, and develop more insights into the neuroprotective activity thereof. In addition to the proposed compounds, their analogues without the nitro group were also synthesized in order to establish the effect of the nitro group on biological activities.

A series of different assays were performed on the synthesized compounds to establish biological activity. All of the compounds were first tested for cytotoxicity. The cytotoxicity profile of these compounds gave an optimum concentration range for a general anti-apoptotic assay to assess the neuroprotective behavior of these compounds. Two more assays were performed in order to evaluate the effect of the synthesized compounds on calcium influx in voltage-gated calcium channels and via the NMDA receptor channel. Compounds were also evaluated for their *S*-nitrosylation ability.

## 2. Results and Discussion

### 2.1. Synthesis

**Scheme 1:**
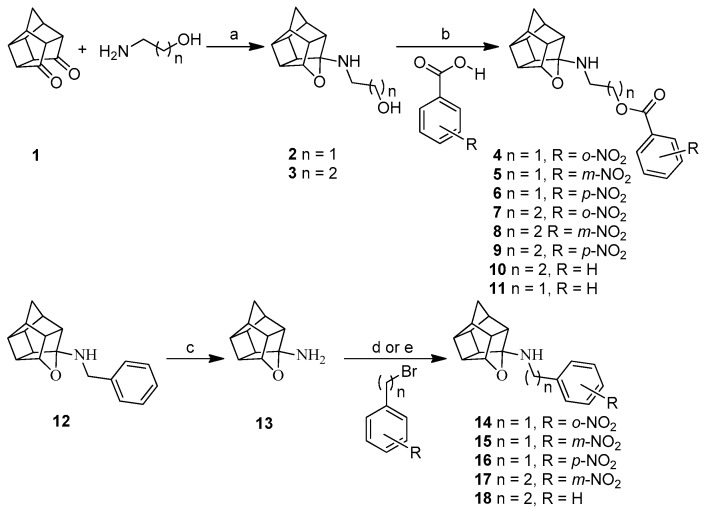
Synthetic routes for targeted compounds. Reagents and conditions: (**a**) 0 °C, THF (dry), MeOH, NaBH_4_; (**b**) 0 °C, DCM (dry), EDC, DMAP; (**c**) 10% Palladium on carbon (Pd/C), C_2_H_5_OH, H_2_ (206 kPa), 50 °C; (**d**) K_2_CO_3_, CH_3_CN, TBAHSO_4_, 70 °C; (**e**) K_2_CO_3_, CH_3_CN, TBAHSO_4_, MW, 250 W, 150 °C, 200 Psi, 3 h.

Pentacyclo[5.4.0.0^2,6^.0^3,10^.0^5,9^]undecane-8-11-dione was synthesized by an earlier reported method [15]. Compounds **4**–**11** were synthesized by using carboxyl activational chemistry to conjugate the amino alcohol derivatives of oxa-pentacycloundecane with nitrobenzoic acids (Scheme 1). The strategy for the synthesis of the amino alcohol derivatives involved reductive amination, where pentacycloundecane dione **1** reacts with amino alcohols such as 2-aminoethanol and 3-aminopropanol followed by reduction with sodium borohydride, which yielded the oxa-bridged pentacycloundecane derivatives **2** and **3** via transannular cyclization. Compounds **14**–**18** were synthesized by nucleophilic substitution (Sn2) of the monoamine cage **13** by appropriate benzyl or phenethyl bromide. The monoamine cage **13** was synthesized by debenzylation of **12** (NGP1-01) under high-pressure catalytic hydrogenation. Compound **12** (NGP1-01) was synthesized by modification of an earlier reported method [16].

**Scheme 2:**
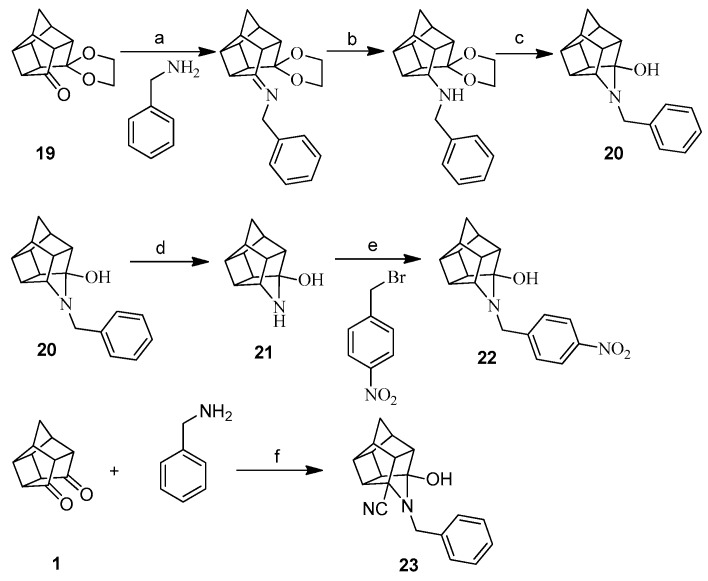
Synthetic routes for aza-hexacyclododecane compounds. Reagents and conditions: (**a**) MW, 150 W, 100 °C, 250 Psi, 1 h; (**b**) EtOH, NaBH_4_; (**c**) Acetone, 4 M HCl; (**d**) 10% Pd/C, C_2_H_5_OH, H_2_ (345 kPa), 50 °C; (**e**) K_2_CO_3_, CH_3_CN, TBAHSO_4_, 70 °C; (**f**) 0 °C, THF (dry), MeOH/AcOH, NaCN.

The synthetic approach for compound **20** (Scheme 2), which is an aza analogue and structural isomer of **12**, was adapted from an earlier reported method [17], with a few variations in reaction conditions. In this approach, the monoprotection of Cookson’s cage compound was first achieved by making a mono ketal cage derivative **19** following the reported procedure [18]. The condensation of this mono ketal cage compound **19** with benzyl amine gave the imine. The reduction of this imine with NaBH_4_, followed by acid hydrolysis, gave the desired aza-bridged compound **20**. The aza compound **22** was synthesized by nucleophilic substitution (Sn2) of the intermediate carbinolamine **21** with *p*-nitrobenzyl bromide, using similar reaction conditions as used for compounds **14**–**18**. Compound **21** was synthesized by debenzylation of **20** using catalytic hydrogenolysis. Compound **23**, the 5-cyano substituted analogue of compound **20**, was prepared by a modification to the procedure described in the Fourie and Snyckers patent [19].

All of the compounds were characterized by ^1^H-, ^13^C-NMR, IR spectra and melting points. The structure of the pentacycloundecane cage moiety was one common feature in all of the structures of the synthesized compounds. This structural component had characteristic ^1^H-NMR signal patterns, which were used unequivocally to establish the presence of the cage component in the final structures. The most characteristic signal for the presence of a cage is the clear AB quartet, because of two unsymmetrical protons on the C-4 bridgehead. This signal appeared at a chemical shift in the range of δ 1.70–1.73 ppm, with a coupling constant generally in the range of 10.6–10.8 Hz.

A typical signal for the presence of an oxa-cage moiety is a triplet at a chemical shift in the range of δ 4.61–4.75 ppm with a coupling constant in the range of 5.0–5.6 Hz. This triplet corresponded to the single proton at the C-11 carbon, and its multiplicity was attributed to the presence of two protons at the adjacent carbons atoms C-1 and C-10. The downfield shift of this methine hydrogen on the C-11 carbon resulted from the deshielding effect of the adjacent oxygen atom. In the case of aza-cage derivatives, this triplet is at a chemical shift in the range of δ 3.21–3.75 ppm, with coupling constants in the range of 4.8–5.0 MHz. This upfield shift, compared with that of its oxa analogues, is attributed to the electronegativity difference between nitrogen and oxygen, and thus, a reduced deshielding effect compared to the oxa derivatives. The same trend was followed by the AB quartet of the protons of the C-4 carbon, although it was less noticeable. The chemical shifts for the rest of the protons in the cage component generally ranged between δ 3.00–2.00 ppm. These signals might appear simply as multiplets or groups of multiplets, apparent quartets, and apparent triplets, depending upon the other structural features in the molecule and the spectrometer frequency of the NMR instrument.

In ^13^C-NMR, in addition to the other aliphatic carbon signals of the cage compound, C-8 and C-11 had distinct chemical shifts. The chemical shift of the C-8 carbon ranged from δ 110 to δ 95 ppm, whereas that of C-11 corresponded to δ 82 ppm. Similar to the proton spectra, the signals in the ^13^C spectra of the aza-cage derivatives also showed the similar upfield shift in comparison to their oxa counterparts, although the difference was more noticeable for the C-8 and C-11 carbons.

In addition to the above-mentioned distinctive NMR signals, the IR spectrum of cage-containing compounds also had a typical IR absorption for sp^3^ hybridised C–H. The absorption for a C–H stretching vibration showed two characteristic peaks at ≈2950 cm^−1^ and ≈2850 cm^−1^, whereas the absorption because of the bending mode of C–H vibration displayed two peaks at ≈1450 cm^−1^ and ≈1350 cm^−1^. The intensity of the two stretching absorptions was always strong, whereas the bending absorption intensity was variable. Generally, the compounds comprising the cage moiety showed a medium intensity for absorptions at ≈1450 cm^−1^; on the other hand, absorptions at ≈1350 cm^−1^ had strong intensity. The above values varied by a few units for different cage-containing compounds, because of the variations in the molecular structure.

After conjugation with the benzoic acid derivatives, the IR spectra of the final compounds also showed an intense absorption peak in the region of 1750–1700 cm^−1^, because of the ester C=O. The broad absorption peak of the OH functional group, which was present in the intermediates, was absent in the conjugated oxa-bridged compounds, as the hydroxyl group had reacted with the carboxylic group to form a new ester linkage. The typical C–H stretching and bending absorptions of the cage structure were retained in the IR spectra of the conjugated compounds. These spectroscopic results corroborated the structures of the final compounds.

Another interesting feature was noted when comparing the methylene –NH-CH_2_− peaks in the ^1^H-NMR of intermediate **3** and its conjugated compounds **7**–**10**. In intermediate **3**, the –CH_2_− attached to –NH appears as a triplet of doublets with *J* = 6.15, 1.3 Hz. Once conjugated to the benzoic acid derivatives, this –CH_2_− peak is shifted downfield, because of the reasons discussed above, but close observation of this peak (after zooming in) shows this triplet of doublets flanked by two small doublets, thus making it an apparent quintet of doublets. This trend is universally observed in the NMR spectra of all of the conjugated compounds (**7**–**10**). It is proposed that the conjugation leads to a fixed conformational feature in the geometry of these molecules, so that the –CH_2_− group attached to the ester functional group comes in close proximity to the –NH-CH_2_− group, leading to the long-range coupling. More investigations are needed to confirm this.

**Figure 3:**
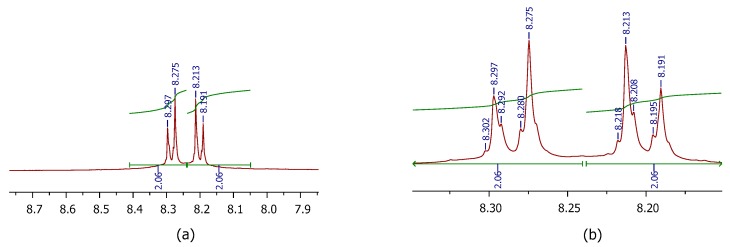
The aromatic region of the ^1^H-NMR spectrum of compound **9** showing the peaks of p-substituted protons. (**a**) The peaks shown as two doublets; (**b**) the peaks as two doublets of triplets, after zooming in the aromatic region.

In the ^1^H spectrum of compound **9**, the *para* substituted aromatic protons showed two doublets at δ 8.27 and 8.20 ppm with *J* = 8.8 Hz, thus indicating a strong coupling from their respective *ortho* protons (Figure 3a). On detailed observation of the aromatic region, these two doublets appeared as a doublet of triplets, with *J* = 2 Hz for each triplet (Figure 3b), which substantiates the long-range coupling of each of the aromatic protons by their respective *meta* and *para* protons, apart from strong coupling from their *ortho* protons.

**Figure 4:**
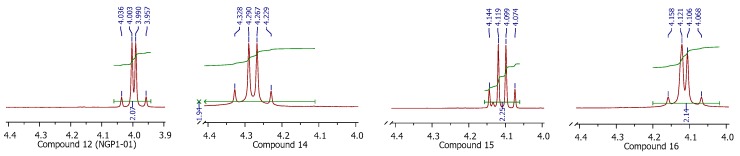
AB quartets of bridging –CH_2_− in the ^1^H-NMR spectra of compounds **12**, **14**, **15,** and **16**.

In the ^1^H spectra of compound **12** (NGP1-01), the –CH_2_− between the cage and aromatic group shows an AB quartet pattern. The same pattern is retained in its structural analogues (**14**–**16**), with –NO_2_ substitution on the phenyl group (Figure 4). It suggests the diastereotopic nature of this –CH_2_− group and the conformational rigidity of such molecules with one carbon linker. The AB quartets of compounds **14**–**16** had a coupling constant of *J* = 15.2 Hz, whereas **12** (NGP1-01) had *J* = 13.2 Hz, which proves that the presence of a substituent on the phenyl group plays a role in deciding the confirmation of such molecules. The greater *J* values of **14**–**16** also indicate that a stronger coupling is experienced by the diastereotopic protons when the –NO_2_ group is present on the phenyl ring. The complete absence of an AB quartet in **17** and **18** supports the compounds with the one carbon linker having such fixed confirmations, as these have a two-carbon linker between the cage and the aromatic group.

### 2.2. Biological Studies

The synthesized compounds were evaluated for their cytotoxicity, neuroprotection, the inhibition of Ca^2+^ influx in ligand-mediated NMDA channels, the inhibition of Ca^2+^ influx in voltage-gated channels, and *S*-nitrosylation ability. This was achieved by different in vitro tests where the PC12 cell line was used for cytotoxicity and neuroprotection studies, and Ca^2+^ studies were performed on synaptoneurosomes. Cytotoxicity assays were performed to assess the toxicity profile of the compounds, and select the optimum concentration range for neuroprotection studies. Neuroprotective studies were done to investigate the anti-apoptotic behavior of the synthesized compounds against neurotoxin-treated PC12 cell lines. Calcium influx studies were conducted to study the inhibitory activity of the synthesized compounds towards voltage-gated calcium channel (VGCC) and *N*-Methyl-d-aspartate receptor (NMDAR)-mediated calcium influx, which is a crucial event in the aetiology of all neurodegenerative diseases. A modified biotin-switch technique (BST) was used to assess the *S*-nitrosylation ability of the compounds with NO-donating moieties.

#### 2.2.1. Cytotoxicity Studies

The MTT proliferation assay [20] was used to assess the cytotoxicity profile of the synthesized compounds. The PC12 cell line [21] from rat adrenal pheochromocytoma was used for the cytotoxicity studies. The cytotoxicity profiles of the compounds were reported in terms of the percentage viability of PC12 cells when treated with respective compounds in a concentration ranging from 1.5625 μM to 200 μM. All of the synthesized compounds showed very good toxicity profiles.

All of the compounds had CC_50_ values (cytotoxic concentration of the compounds to cause death to 50% of the viable cells) of more than 200 µM. Some of the compounds showed more than 100% cell viability (Table 1); this might be because those compounds, on treatment of the cells, increased the activity of the succinate dehydrogenase enzyme within the mitochondria without affecting the cell viability. Some of these compounds might be preventing baseline apoptosis. Another reason can be the natural variations in the cellular metabolism.

The introduction of a nitro group did not affect the cytotoxicity profiles of the molecules significantly. It was also observed that the position of substitution of the nitro on the phenyl group did not play any significant role towards the cytotoxicity of the compounds.

The highest concentration where the compounds did not show any cytotoxicity was 50 µM; therefore, this concentration was selected as the maximum concentration for the neuroprotection studies.

#### 2.2.2. Neuroprotection Studies

In the neuroprotection assay, the cells were stressed by paraquat (1,1-dimethyl-4,4-bipyrimidyl chloride) as a toxin to model neurodegeneration. A dose-dependent cytotoxicity response of paraquat towards PC12 cells was established to determine a suitable concentration and incubation time to model neuroprotection. At a concentration of 250 μM and a treatment period of 24 h, paraquat induced approximately 30% cell death, while pre-treatment with *N*-acetyl-cysteine (NAC) (500 μM) restored the cell viability to 85%. Cell viability was determined using the CellTiter-Blue reagent (Promega). The neuroprotective potential of the test compounds was determined at three concentrations, i.e., 5 μM, 25 μM, and 50 μM, by treating PC12 cells with the neurotoxin paraquat at a concentration of 250 μM. NAC was used as a positive control for each experiment to ensure that cell death was induced at a level that is at least partially restorable.

Most of the compounds showed very good neuroprotection when compared with NAC. It is to be noted that in all of the tests conducted to study neuroprotection, the concentration of the positive control NAC was 500 μM, whereas the maximum concentration used for the test compounds was 50 μM. The NAC functions as a precursor for glutathione (GSH) synthesis, and thus protects against oxidative stress as an antioxidant [22]; as such, it may become rapidly exhausted through paraquat-induced oxidative stress. Consequently, there is a need for a continuous supply to provide sufficient protection. The relatively high concentrations of NAC required to afford neuroprotection may also relate to the enzymatic process that is necessary for the biosynthesis of GSH. Excessive concentrations of extracellular NAC shifts the equilibrium, for both its transport into the cell as well as the enzymatic pathway required for the synthesis of GSH. These features together necessitate a relatively high concentration of NAC to be used in order to provide significant protection. Unless the mechanism of action of the test compounds is assumed to be similar to that of NAC, it is not possible to make a direct comparison in terms of efficacy. In this assay, most of the test compounds showed dose-dependent neuroprotection behavior, although some of the compounds had a negative percentage of neuroprotection at one of the concentrations tested.

The comparison of neuroprotection results of the compounds without any nitro group (**11**, **10**, **12**, and **20**) with those of their analogues with a nitro group (**6**, **9**, **16**, and **22**, respectively) shows that compounds with the nitro functional group have more neuroprotective ability against the neurotoxin used (Table 2). The two compounds (**10** and **9**) that have a three-carbon linker between the amine and ester groups were the exception. However, the difference in the neuroprotection caused by compounds **10** and **9** was statistically non-significant.

At a 50-μM concentration of the test compounds, compound **11** showed 1.38% neuroprotection, whereas its analogous structure with the nitro functional group (**6**) had 26.55% neuroprotection. Similarly, compound **12** showed 3.38% neuroprotection, and its nitro analogue (**16**) showed 18.71% neuroprotection. These results suggest that the presence of a NO-donating moiety in the structure of the molecule enhances its neuroprotective ability, but simultaneously, the other features of the molecule also contribute towards its neuroprotection profile. The analysis of the neuroprotection of different positional isomers also suggested that the position of the nitro functional group on the phenyl ring of the molecule does not influence its neuroprotective ability.

In the neurotoxicity model used in these tests, paraquat, an inducer of oxidative stress, was used as the neurotoxin. It is possible that not all of the neuroprotective compounds would necessarily be able to counteract the effects of paraquat, due to their specific mechanisms of action. Interestingly, compound **12** (NGP1-01), which shows neuroprotective properties through NMDA and L-type voltage-operated calcium channels [23,24], exhibited only 3.38% neuroprotection at 50 μM under the test conditions. It might show a better neuroprotection against other neurotoxins with different neurotoxic mechanisms. It is also interesting to note that compound **12** gave better and more statistically significant results at 25 μM concentration, but not at 50 μM. Compound **5** also showed better neuroprotection at 25 μM than at 50 μM, although it was with larger standard deviation. The better neuroprotection at a lower concentration can be because these compounds became toxic to the stressed cells at the higher concentrations. The same can be applied to the other compounds (**7**, **11,** and **18**) that showed the highest neuroprotection at 5 μM concentration, followed by neuroprotection at 25 μM concentration, under given test conditions. Compounds **7**, **11**, and **18** showed 14.53%, 14.25% and 12.72% neuroprotection at 5 μM versus 5.98%, 9.37%, and 5.52% at 25 μM, respectively. The larger standard deviations in some of the results can be attributed to the nature of this neuroprotection assay, where unhealthy cells (as a result of the exposure to the neurotoxin) were used. Such biological assays are more prone to variations, especially when attempting to reverse cell death. 

#### 2.2.3. NMDA-Mediated Ca^2+^ Studies

**Figure 5:**
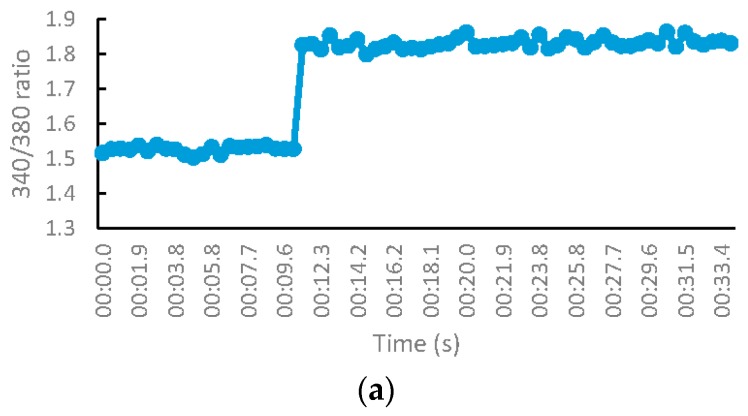

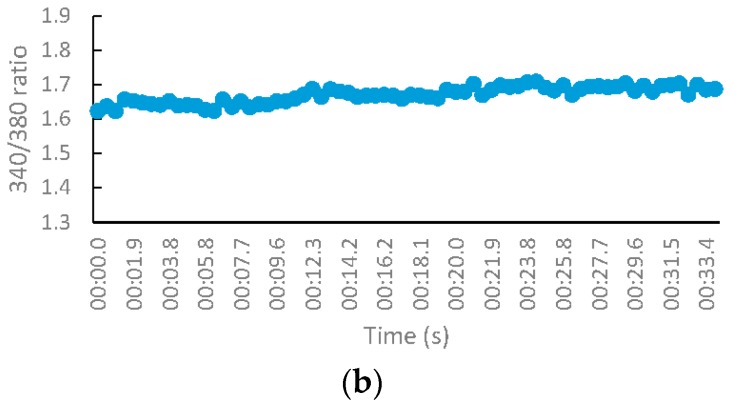
Fluorescence profile of synaptoneurosomes in Fura-2/AM experiments for studying the *N*-methyl-d-aspartate (NMDA)-mediated Ca^2+^ influx. (**a**) Stimulation at 10.6 s by stimulating buffer in the absence of any test compound; (**b**) Stimulation at 11.1 s by stimulating buffer in the presence of a known NMDA receptor (NMDAR) blocker (MK-801).

The NMDA receptor (NMDAR) activity of the test compounds was evaluated on murine synaptoneurosomes using the fluorescent ratiometric calcium indicator, Fura-2/AM. Pure DMSO was used as the control. MK-801 and NGP1-01 were used as positive controls. Synaptoneurosomes consisted of the presynaptic terminal, including mitochondria and synaptic vesicles, with the postsynaptic membrane and the postsynaptic density proteins [25]. These were obtained by the homogenization and fractionation of the rat brain cortex. The resealed vesicles or isolated terminals break away from the axon terminals during the homogenization of the cortical tissues. The synaptoneurosomes retain the pre- and postsynaptic properties, which makes them useful for the study of synaptic transmission. The molecular machinery used in neuronal signaling is also retained in the synaptoneurosomes, which are capable of the uptake, storage, and release of neurotransmitters [26]. Freshly isolated synaptoneurosomes were loaded with Fura-2/AM by incubation in a calcium-free buffer, so that the membrane-permeable Fura-2/AM enters the synaptoneurosomes. These synaptoneurosomes were then suspended in a calcium-containing buffer. NMDARs were activated by injecting a NMDA/glycine stimulation buffer, thus leading to NMDA-mediated calcium influx. The synaptoneurosomes exhibited the desirable physiological function, as per their fluorescence profile, when injected with the stimulation buffer, both in the absence of any test sample and in the presence of known NMDAR blocker MK-801 (Figure 5). The changes in the fluorescence intensity were measured before and after the activation of NMDAR. The effect of the test compounds on this calcium influx was measured by monitoring the changes in the fluorescence intensities.

**Figure 6:**
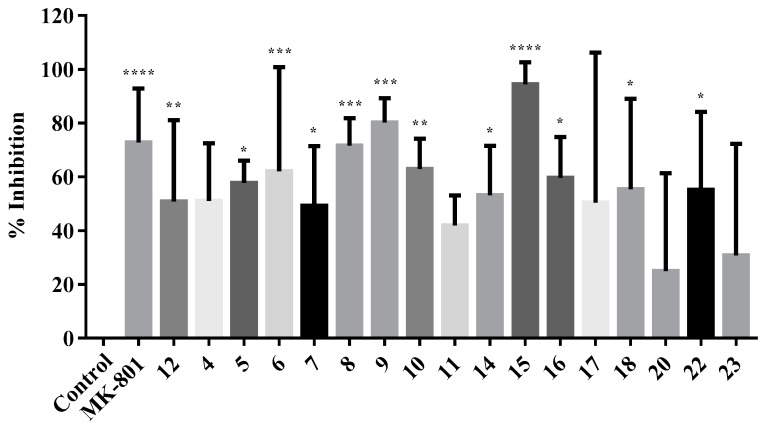
Screening of test compounds (100 μM) for their inhibitory effect on NMDA-mediated calcium influx using murine synaptoneurosomes. Each bar represents the mean percentage of inhibition and standard deviation. The asterisks indicate the statistical significance (*, *p* < 0.05; **, *p* < 0.005; ***, *p* < 0.001 and ****, *p* < 0.0001) when compared to the zero inhibition control.

All of the compounds were investigated for their inhibitory effect on NMDA receptor-mediated calcium influx at a concentration of 100 μM. The percentage of the inhibition of calcium influx obtained after the statistical analysis of raw data is depicted in Figure 6 for all of the compounds under investigation. MK-801 and compound **12** (NGP1-01) were used as positive controls at a concentration of 100 μM. This concentration was selected as approximately 80% inhibition of calcium flux was observed for the positive control MK-801, and it was shown to be an effective concentration for the screening of polycyclic structures in synaptoneurosomal preparations [23,27]. Pure DMSO in place of test samples was used as the control.

All of the tested compounds showed moderate to good inhibition of NMDA-mediated calcium influx. The control, which had DMSO in place of a test sample solution, had 0% inhibition. The positive controls MK-801 and compound **12** (NGP1-01) showed 72.77% and 50.84% inhibition, respectively. To examine the effect of the presence of the nitro group on the NMDA-mediated calcium influx, a comparative study of the percentage inhibition of the compounds without any nitro group on the phenyl ring (compounds **11**, **10**, and **12**) was done with their analogues with nitro groups at any of the *ortho*, *meta*, or *para* positions of the phenyl group. All of the compounds with the nitrophenyl group showed better inhibitory activity than their respective parent compounds without the nitro substitution, although the difference in the activities of non-nitro and nitro compounds was not statistically significant. Compound **7**, which is an *ortho*-nitro analogue of compound **10**, was an exception to the above trend, and exhibited 49.26% inhibition, whereas compound **10** showed 62.92%. Aza-compound **22** with *p*-nitrophenyl also showed better inhibition (55.22%) of NMDA-mediated calcium flux than compound **20** (24.92%), which is its analogue without any nitro group on the phenyl ring.

**Figure 7:**
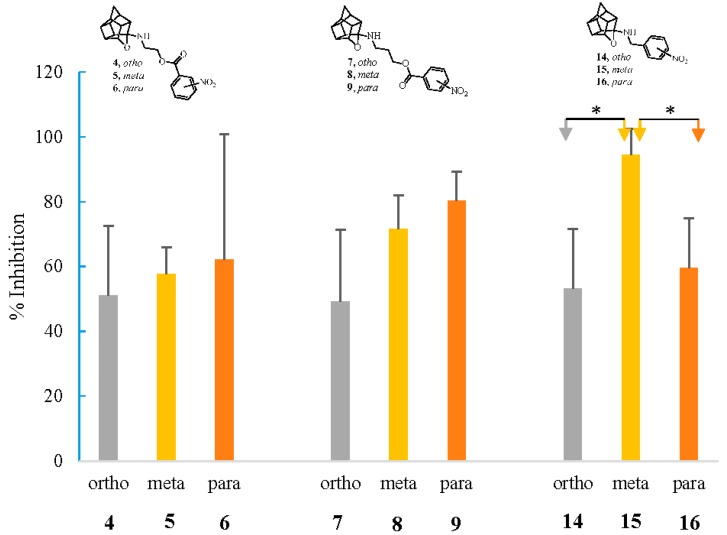
Percentage inhibition of NMDA-mediated calcium influx by three different sets of *o*-, *m*- and *p*-position isomers of compounds *viz*. **8**, **7** and **4**; **10**, **9** and **11**; and **17**, **18** and **16**, at a test concentration of 100 μM. The statistical significance of the difference between the two percentage inhibition values is determined by the *t*-test, and it is indicated as: *, *p* < 0.05.

From the study of percentage inhibition of NMDA-mediated calcium influx of different *o*-, *m*-, and *p*-nitro positional isomers, it was observed that the position of the nitro group on the phenyl group in the test compounds also plays a role in their inhibitory activities. The inhibitory activity of the three different sets of *o*-, *m*-, and *p*-nitro positional isomers (**4**, **5**, and **6**; **7**, **8**, and **9**; and **14**, **15**, and **16**; respectively) is shown in Figure 7. In the sets of **4**, **5**, and **6** and **7**, **8**, and **9**, the order of inhibitory activity followed by the compounds is *para* > *meta* > *ortho*, where the geometrical symmetry of the *p*-nitro compounds could be the reason for the better inhibitory activity. In the *o*-, *m*-, and *p*-positional isomer set of **14**, **15,** and **16**, the *m*-nitro isomer (**15**) has the highest activity. It is interesting to note that this set of compounds **14**, **15**, and **16** has an effective two-bond distance between the cage amine and the nitro phenyl compared to the five and six-bond distance in the former two sets. As discussed in Section 2.1, in the ^1^H spectra of these compounds (**14**, **15**, and **16**), the bridging –CH_2_− between the cage and the aromatic group showed an AB quartet pattern, suggesting the diastereotopic nature of the bridging –CH_2_− group, and thus the conformational rigidity of such molecules with one carbon linker. The conformational rigidity of these compounds might thus be a more dominating factor than the symmetry factor in leading to the *para* > *meta* > *ortho* rank order of the other sets of compounds (**4**, **5**, and **6** and **7**, **8**, and **9**).

In comparison of the activity of oxa- and aza-pentacycloundecane derivatives, the oxa-compounds **12** and **16** showed slightly better, though not significant, NMDAR inhibitory activity (50.84% and 59.6%, respectively) than their aza counterparts **20** and **22** (24.92% and 55.22%, respectively).

#### 2.2.4. Voltage-Gated Ca^2+^ Studies

**Figure 8:**
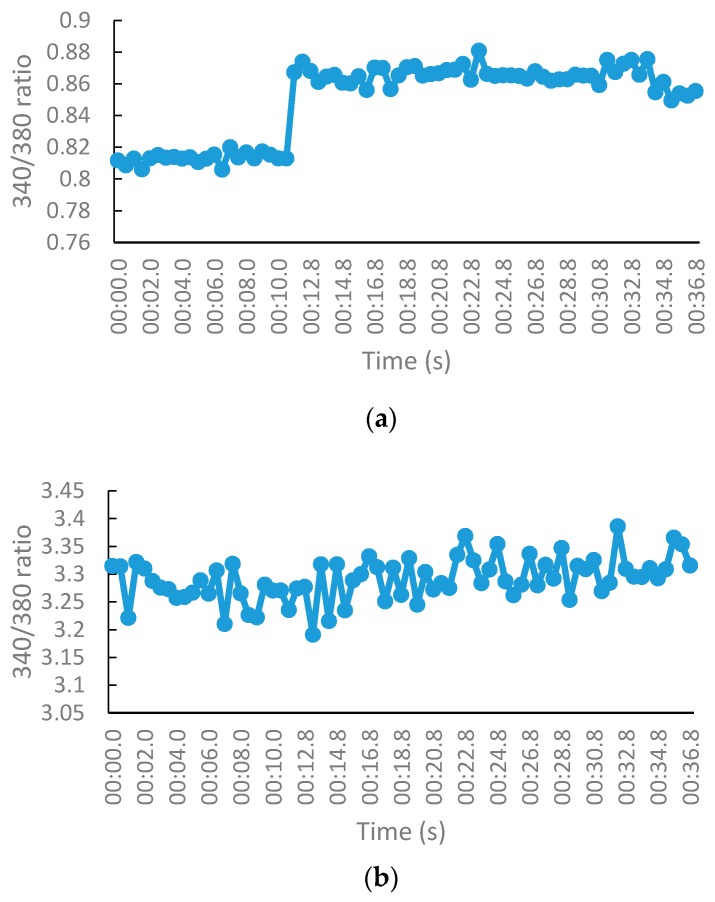
Fluorescence profile of synaptoneurosomes in Fura-2/AM experiments for studying the voltage-gated Ca^2+^ influx. (**a**) Stimulation at 11.1 s by depolarization buffer in the absence of any test compound; (**b**) Stimulation at 11.1 s by depolarization buffer in the presence of a known voltage-gated calcium channel (VGCC) blocker (nimodipine).

The effect of the test compounds on VGCC was evaluated on murine synaptoneurosomes with the same methodology as that for NMDA-mediated Ca^2+^ studies using the fluorescent ratiometric calcium indicator, Fura-2/AM. DMSO was used as the control, whereas nimodipine and NGP1-01 were used as positive controls, at a concentration of 100 μM. At physiologic or resting membrane potential, VGCCs are closed. These channels were opened by injecting the depolarization buffer (containing 140 mM of K^+^), and thus allowing VGCC-mediated calcium influx into the cells, which resulted in an increase in the fluorescent ratio in the presence of calcium. The fluorescence profile of synaptoneurosomes, when injected with the depolarization buffer in the absence of any test sample and in the presence of known VGCC blocker nimodipine, is displayed in Figure 8.

**Figure 9:**
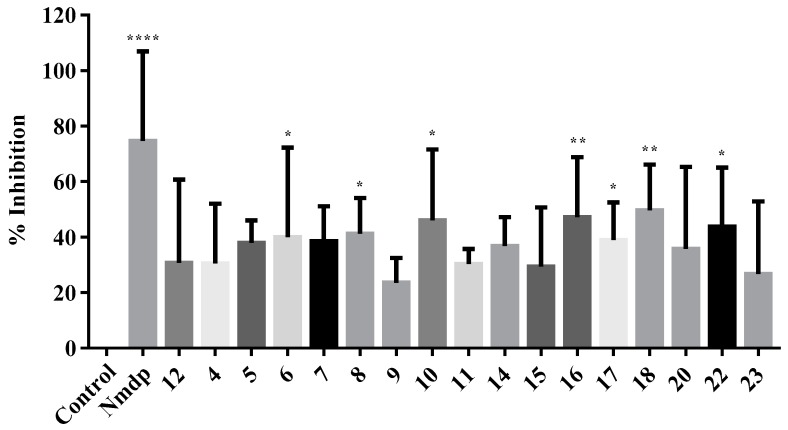
Screening of test compounds (100 μM) for their voltage-gated calcium influx using murine synaptoneurosomes. Each bar represents mean percentage of inhibition and standard deviation. The asterisks indicate the statistical significance (*, *p* < 0.05; **, *p* < 0.005 and ****, *p* < 0.0001) when compared to the control.

The fluorescence intensity was measured before and after the activation of VGCC. The effect of the test compounds on this calcium influx was measured by monitoring the changes in the fluorescence intensities. The screening results of the test compounds are depicted in Figure 9.

The compounds under investigation showed low to moderate voltage-gated calcium influx inhibition. The control, which had DMSO only in place of the test sample solution, had 0% inhibition. The positive controls nimodipine and compound **12** (NGP1-01) showed 74.64% and 30.71% inhibition, respectively, although the latter value was not statistically significant. To study the effect of the presence of the nitro group on the voltage-gated calcium influx, the percentage inhibition of **10**, **11**, and **12** (the compounds without any nitro group on the phenyl ring) was compared with their analogues with a nitro group at any *ortho*, *meta*, or *para* position of the phenyl group.

With a few exceptions, the introduction of a nitro group enhanced the percentage inhibition of voltage-mediated calcium influx. All of the *ortho*, *meta*, and *para* nitro-substituted variants of **11** had better percentage inhibitions of calcium influx, although these values were statistically non-significant.

Another interesting inference that can be drawn from above data is that the *para*-nitro positional isomer always had the highest percentage inhibition, barring the exceptional case of the set of *ortho*, *meta*, and *para*-substituted variants of compound **10**. This indicates the *para* position as the more favorable nitro substitution position for the inhibition of voltage-mediated calcium influx among this series of compounds.

The voltage-gated calcium activity of the oxa compounds **12** and **16** (30.71% and 47.15%) was minimally different from their aza analogues **20** and **22** (35.69% and 43.76%), respectively.

#### 2.2.5. *S*-Nitsosylation Studies

For evaluating the *S*-nitrosylation ability of the test compounds, the biotin switch technique (BST) was implemented, with some experimental modifications from the original reported method [28]. The rat brain homogenate was substituted with cysteine–glycine dipeptide. The ultraviolet absorbance of pyridine-2-thione at 343 nm, which was used to detect *S*-nitrosylation in the reported method, was also employed to quantify the *S*-nitrosylation ability. After *S*-methylthiolation, ascorbate was not added for nitrosothiol decomposition, considering the spontaneous decomposition of nitrosothiol before the *N*-[6-(biotinamido)hexyl]-3′-(2′-pyridyldithio)propionamide (biotin-HPDP) addition [28,29]. The cysteine thiols were allowed to react with equimolar quantities of NO donors by mixing the two in a 1:1 stoichiometric ratio in HEN buffer. The mixture was then incubated with methyl methanethiosulfonate (MMTS) to block the free thiols that had been left after treatment with the NO donor. Acetone was used to precipitate the excess MMTS. Biotin-HPDP was added to form a *S*-biotinylated product from the free thiols generated by the spontaneous nitrosothiol decomposition. The amount of the by-product, pyridine-2-thione, of this reaction was estimated by the absorbance at 343 nm, which was directly proportional to the amount of biotin-HPDP used, and hence to the extent of *S*-nitrosylation. The average ultraviolet absorbance at five-minute intervals for 15 min into the reaction was measured, and the *S*-nitrosylation potency was expressed as the percentage relative to the control of each experimental run. All of the compounds with nitro groups were used for the *S*-nitrosylation study. Sodium nitroprusside (SNP) and 2-(*N*,*N*-diethylamino)-diazenolate 2-oxide sodium salt hydrate (DEA/NONOate) were used as positive controls, whereas compound **12** (NGP1-01) was used as a negative control.

**Figure 10:**
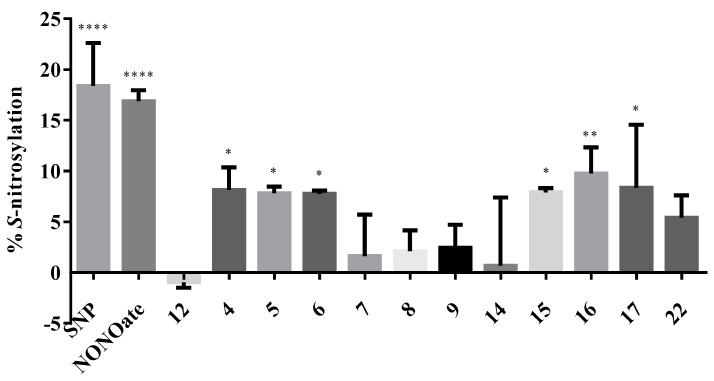
Percentage *S*-nitrosylation caused by the test compounds relative to the control. The test was conducted with equimolar quantities of the test samples and the glycine–cysteine dipeptide. Each bar represents the mean percentage *S*-nitrosylation and standard deviation. The asterisks indicate the statistical significance (*, *p* < 0.05; **, *p* < 0.005 and ****, *p* < 0.0001) when compared to the control.

The extent of *S*-nitrosylation caused by each compound is expressed as the percentage relative to that of the control, and is depicted in Figure 10. The positive controls SNP and DEA/NONOate showed 18.39% and 16.87% *S*-nitrosylation of the cysteine thiol residue of the cysteine–glycine dipeptide. As expected, the negative control **12** (NGP1-01), which did not have any NO-donating group, showed no S-nitrosylation (experimental value of −0.96%). The test compounds showed low to good S-nitrosylation ability compared to the positive controls.

No significant difference in *S*-nitrosylayion ability was observed for the *ortho*, *meta*, and *para* positional isomers. However, the linker between the cage and NO-donating moiety seemed to play a more important role for nitrosylation ability. Compounds **16** and **15**, with a one-carbon hydrocarbon linker (two bond distance) between the cage amine and the nitro phenyl group, showed the highest *S*-nitrosylation percentages of 9.77% and 7.89%, respectively. Compound **14** was an exception with the one-carbon linker, and had a very low *S*-nitrosylation ability, which was probably because of the steric hindrance in the conformationally restricted structure. The *S*-nitrosylation potency of compounds **4**, **5**, and **6**, with a two-carbon ester linkage (five-bond distance) between the cage amine and nitrophenyl, were second highest with the values of 8.14%, 7.81% and 7.76%, respectively. The compounds with a three-carbon ester linkage (six-bond distance) showed the lowest *S*-nitrosylation, with 2.42%, 2.09%, and 1.61% for compounds **9**, **8**, and **7**, respectively. The percentage *S*-nitrosylation by the compound **17**, with a 2C-hydrocarbon linker, was 8.34%, whereas its one-carbon analogue **15** caused 7.89% *S*-nitrosylation. It can be seen that for determining the *S*-nitrosylating ability, the steric and conformational factors resulting from the bond distance and the position of the nitro group on the phenyl ring are more critical than the electronic effects generated by the presence or absence of an ester linkage. The *S*-nitrosylation with the aza compound **22** was 5.40%, whereas its oxa analogue showed a slightly higher *S*-nitrosylation ability (9.77%).

The standard deviation observed in these experiments was significantly smaller than that obtained in the assays described in previous sections. This is because of the biochemical nature of these assays, where the test conditions are more controlled than in the biological assays.

## 3. Materials and Methods

### 3.1. Synthesis

#### 3.1.1. General Information

Unless otherwise specified, all of the chemicals were obtained from Sigma-Aldrich or Merck. All of the chemicals were of analytical grade, and used without further purification unless specified. To obtain dry THF, commercially available THF was pre-dried over 3Å molecular sieves before distilling over sodium wire and a small quantity of benzophenone. To get dry DCM, commercially available DCM was initially dried over anhydrous calcium chloride, and then distilled. The distillate was collected and stored in an amber glass bottle away from light over 3Å molecular sieves.

The structures of the compounds were characterized by using nuclear magnetic resonance (NMR), Fourier transform infrared (FT-IR), and mass spectrometry (MS) techniques. In the NMR spectroscopy, ^1^H, ^13^C, and distortionless enhancement of polarization transfer (DEPT) analysis were performed for structural elucidation. The carbon peaks in the ^13^C-NMR spectroscopy were further differentiated as –CH_3_, CH, or CH_2_ carbons by DEPT-135 analysis, where CH_3_ and CH gave positive signals, and CH_2_ gave a negative signals.

NMR’s were obtained using Varian 600 MHz, Varian 400 MHz, Bruker 400 MHz, and Varian 200 MHz instruments. The chemical shifts were reported as δ values in ppm downfield from tetramethylsilane (TMS) and deuterated residual solvent peaks as internal standards (δH, CDCl_3_ 7.26 ppm; δC, CDCl_3_ 77.16 ppm). The coupling constants (*J*) are given in hertz (Hz), and the multiplicities of NMR signals are expressed as: s, singlet; brs, broad singlet; d, doublet; t, triplet; q, quartet; quin, quintet; m, multiplet; ABq, AB quartet; dd, doublet of doublets; td, triplet of doublets; dtd, doublet of triplet of doublets; appt, apparent triplet. The ^1^H-NMR data is presented as follows: chemical shift in ppm (multiplicity, coupling constant, integration as number of protons). IR spectra were recorded using a Perkin Elmer Spectrum 400 FT-IR/FT-NIR spectrometer with an attenuated total reflectance (ATR) attachment. Mass spectra were recorded by Perkin Elmer MS with a Flexar SQ 300 MS detector using direct infusion electrospray ionization mass spectrometry. Melting points were measured using the glass capillary method. The melting points were determined using a Stuart SMP-10 melting point apparatus. All of the melting points were uncorrected.

The physical characteristics of the compounds are reported as physical state, color, and melting point (m.p.).

#### 3.1.2. General Procedure for the Synthesis of Amino Alcohol Derivatives (**2**,**3**)

Pentacyclo[5.4.0.0^2,6^.0^3,10^.0^5,9^]undecane-8-11-dione **1** (5 g, 28.7 mmol) was dissolved in dry tetrahydrofuran (50 mL) in a 250-mL round-bottom flask. The round-bottom flask was kept in an external ice bath with stirring, so as to maintain the temperature below 3 °C. Amino alcohol (28.7 mmol) was added drop-wise using a dropping funnel, while stirring at a lower temperature. The carbinolamine started to precipitate within 10 min, and the mixture was stirred for another 20 min, after which dry methanol (30 mL) was added, and the mixture was removed from the ice bath. Once all of the carbinolamine precipitate was dissolved, sodium borohydride (1.5 g) was added slowly, and the mixture was stirred at room temperature overnight. The reaction mixture was concentrated in vacuo, water was added, and it was followed by extraction with dichloromethane (4 × 50 mL). Anhydrous sodium sulphate was used to dry the combined organic fractions, which on evaporation gave a yellow oil. The mixture was purified using column chromatography (10% ethanol in ethyl acetate).

*8-(2-Aminoethanol)-8,11-oxapentacyclo[5.4.0.0^2,6^.0^3,10^.0^5,9^]undecane* (**2**). A thick transparent liquid, yield: 57.94%. FT-IR (ATR): ν_max_ (cm^−1^) = 3312.65, 2958.19, 2862.28, 1662.76, 1455.82. ^1^H-NMR (200 MHz, CDCl_3_): δ 4.62 (t, *J* = 5.2 Hz, 1H), 3.60–3.56 (m, 2H), 3.02–2.98 (m, 2H), 2.85–2.65 (m, 4H), 2.61–2.40 (m, 4H), 1.72 (ABq, *J* = 10.6 Hz, 2H). ^13^C-NMR (75 MHz, CDCl_3_): δ 97.5, 82.39, 63.60, 55.13, 54.79, 46.51, 44.91, 44.62, 44.32, 43.29, 43.15, 41.92, 41.55. MS (ESI) *m*/*z*: 219.99 (M + H^+^), 221.01, 222.02.

*8-(3-Aminopropanol)-8,11-oxapentacyclo[5.4.0.0^2,6^.0^3,10^.0^5,9^]undecane* (**3**). A white waxy solid, yield: 35.22%, m.p.: 59–61 °C. FT-IR (ATR): ν_max_ (cm^−1^) = 3169.13, 2956.34, 2862.65, 1664.21, 1509.79. ^1^H-NMR (200 MHz, CDCl_3_): δ 4.64 (t, *J* = 5.2 Hz, 1H), 3.79 (t, *J* = 5.4 Hz, 2H), 3.05–2.98 (m, 2H), 2.88–2.68 (m, 8H), 1.72 (ABq, *J* = 10.6 Hz, 2H), 1.77–1.66 (m, 2H). ^13^C-NMR (75 MHz, CDCl_3_): δ 104.60, 82.72, 63.26, 55.41, 54.86, 44.93, 44.89, 44.66, 44.40, 43.10, 42.96, 41.89, 41.63, 32.34. MS (ESI) *m*/*z*: 234.28 (M + H^+^), 235.22, 236.24.

#### 3.1.3. Procedure for Synthesis of Aza-Bridged Compounds **20**

##### 4-Benzyl-4-azahexacyclo[5.4.1.0^2,6^.0^3,10^.0^5,9^.0^8,11^]dodecan-3-ol (**20**)

A solution of mono ketal cage **19** (0.7 g, 3.20 mmol) and benzyl amine (0.344 g, 3.21 mmol) in 2.5 mL ethanol was reacted in a microwave reactor (µwave = 150 W, T = 100 °C, P = 250 Psi) in a sealed 10-mL microwave vessel. Progress of the reaction was monitored by TLC. After the completion of the reaction (TLC, 1 h), NaBH_4_ (0.243 g, 6.42 mmol) was added portion-wise to the cooled solution, and the mixture was stirred at room temperature for 8 h. The solution was then concentrated in vacuo, water (10 mL) was added, and the crude reaction mixture was extracted with dichloromethane (3 × 10 mL). The combined organic extract was washed with brine (10 mL), dried with anhydrous Na_2_SO_4_, and the solvent was evaporated in vacuo. To the reaction mixture obtained after the evaporation of the solvent, acetone (20 mL) and aq. 4 M HCl (15 mL) were added. The mixture was stirred at room temperature for 18 h. After stirring, water (150 mL) was added, and the solution was basified to pH 12–13 with aq. 1 M NaOH, and extracted with dichloromethane (4 × 30 mL). The combined organic extract was dried with anhydrous Na_2_SO_4_ and concentrated to obtain a crude reaction mixture. The final product was obtained by column chromatography using a mobile phase made of 40% ethanol and 60% ethyl acetate as a white crystalline solid, yield: 43.71%, m.p.: 160–162 °C. FT-IR (ATR): ν_max_ (cm^−1^) = 3111.11, 3057.35, 2974.44, 2968.25, 2869.19, 2835.05, 1605.29, 1497.46. ^1^H-NMR (200 MHz, CDCl_3_): δ 7.28–7.13 (m, 5H), 3.68 (brs, 1H, OH), 3.55 (ABq, *J* = 14.9 Hz, 2H, NCH_2_) 3.21 (t, *J* = 4.9 MHz, 1H), 2.90–2.40 (m, 7H), 2.28 (t, *J* = 4.6, 1H), 1.52 (ABq, *J* = 10.4 Hz, 2H). ^13^C-NMR (75 MHz, CDCl_3_): δ 139.29, 128.47, 128.32, 126.74, 64.77, 55.05, 51.65, 50.67, 45.49, 44.59, 42.89, 42.28, 41.62, 41.28. HRMS (ESI/TOF) *m*/*z*: [M + H]^+^ Calcd. for C_18_H_20_NO 266.1545; Found 266.1539.

#### 3.1.4. Procedure for Synthesis of Oxa-Bridged Compounds (**4**–**11**)

##### 2-(8,11-Oxapentacyclo[5.4.0.0^2,6^.0^3,10^.0^5,9^]undecane-8-amino)ethyl 2-nitrobenzoate (**4**) 

A solution of 8-(2-aminoethanol)-8,11-oxapentacyclo[5.4.0.0^2,6^.0^3,10^.0^5,9^]undecane **2** (0.92 g, 4.20 mmol) in dry dichloromethane (30 mL) was cooled down to 0 °C in an external ice bath. 1-Ethyl-3-(3′-dimethylamino)carbodiimide (0.65 g, 4.20 mmol), 3-nitrobenzoic acid (0.70 g, 4.19 mmol), and 4-(dimethylamino)-pyridine (0.05 g, 0.41 mmol) were added while stirring in an ice bath. After 20 min, the reaction was removed from the ice bath and stirred for 72 h at room temperature. The progress of the reaction was monitored by TLC. After completion of the reaction, a saturated solution of sodium bicarbonate (30 mL) was added, followed by extraction with dichloromethane (4 × 20 mL). Anhydrous sodium sulphate was added to the combined organic fractions to remove traces of water after extraction. The solvent was evaporated under reduced pressure to give the crude reaction mixture. The final product was purified from the crude reaction mixture by column chromatography using a mobile phase made of different proportions of ethyl acetate in hexane as a brownish yellow and transparent thick liquid, yield: 38.86%. FT-IR (ATR): ν_max_ (cm^−1^) = 3329.55, 2964.04, 2864.74, 1729.94, 1649.84. ^1^H-NMR (200 MHz, CDCl_3_): δ 7.92–7.89 (m, 1H), 7.77–7.61 (m, 3H), 4.64 (t, *J* = 5.6 Hz, 1H), 4.44 (t, *J* = 5.8 Hz, 2H), 3.14 (t, *J* = 5.8 Hz, 2H), 2.83–2.38 (m, 8H), 1.71 (ABq, *J* = 10.6 Hz, 2H). ^13^C-NMR (101 MHz, CDCl_3_) and DEPT-135: δ 165.35 (C=O), 148.20 (ArC), 132.90 (ArCH), 131.80 (ArCH), 130.05 (ArCH), 127.57 (ArC), 123.84 (ArCH), 110.91 (C-8), 82.39 (C-11), 66.95 (CH_2_O), 55.19 (CH), 54.74 (CH), 44.82 (CH), 44.53 (CH), 44.50 (CH), 43.22 (CH_2_NH), 43.10 (CH), 42.27 (CH_2_), 41.89 (CH), 41.50 (CH). HRMS (ESI/TOF) *m*/*z*: [M + H]^+^ Calcd. for C_20_H_21_N_2_O_5_ 369.1450; Found 369.1448.

The synthesis of compounds (**5**, **6**, and **11**) was carried out using amino alcohol derivative **2**, whereas that of compounds (**7**–**10**) was done using the amino alcohol derivative **3**, and following the similar procedure as described for compound **4**.

*2-(8,11-Oxapentacyclo[5.4.0.0^2,6^.0^3,10^.0^5,9^]undecane-8-amino)ethyl 3-nitrobenzoate* (**5**). A brownish yellow thick liquid, yield: 48.67%. FT-IR (ATR): ν_max_ (cm^−1^) = 2964.90, 2864.01, 1722.14, 1616.68, 1531.00. ^1^H-NMR (200 MHz, CDCl_3_): δ 8.86 (t, *J* = 1.8 Hz 1H), 8.45–8.34 (m, 2H), 7.65 (t, *J* = 8 Hz, 1H), 4.64 (t, *J* = 5.6 Hz, 1H), 4.46 (t, *J* = 5.6 Hz, 2H), 3.22 (t, *J* = 5.8 Hz, 2H), 2.85–2.42 (m, 8H), 1.72 (ABq, *J* = 10.6 Hz, 2H). ^13^C-NMR (101 MHz, CDCl_3_) and DEPT-135: δ 164.52 (C=O), 148.26 (ArC), 135.36 (ArCH), 131.98 (ArC), 129.64 (ArCH), 127.43 (ArCH), 124.63 (ArCH), 110.01 (C-8), 82.53 (C-11), 66.50 (CH_2_O), 55.31 (CH), 54.76 (CH), 44.85 (CH), 44.68 (CH), 44.57 (CH), 43.29 (CH_2_NH), 43.10 (CH), 42.52 (CH_2_), 41.89 (CH), 41.54 (CH). HRMS (ESI/TOF) *m*/*z*: [M + H]^+^ Calcd. for C_20_H_21_N_2_O_5_ 369.1450; Found 369.1443.

*2-(8,11-Oxapentacyclo[5.4.0.0^2,6^.0^3,10^.0^5,9^]undecane-8-amino)ethyl 4-nitrobenzoate* (6). A light yellow solid, yield: 41.67%, m.p.: 107–109 °C. FT-IR (ATR): ν_max_ (cm^−1^) = 3314.22, 2968.31, 2866.05, 1714.36, 1610.57, 1527.62. ^1^H-NMR (200 MHz, CDCl_3_): δ 8.27 (dd, *J* = 7.0 Hz, 1.8 Hz, 4H), 4.63 (t, *J* = 5 Hz, 1H), 4.46 (t, *J* = 5.6 Hz, 2H), 3.21 (t, *J* = 5.6, 2H), 2.87–2.43 (m, 8H), 1.72 (ABq, *J* = 10.6 Hz, 2H). ^13^C-NMR (75 MHz, CDCl_3_): δ 164.76, 150.64, 135.70, 130.84, 123.61, 109.35, 82.71, 66.65, 55.51, 54.86, 44.95, 44.82, 44.66, 43.41, 43.17, 42.56, 42.02, 41.64. HRMS (ESI/TOF) *m*/*z*: [M + H]^+^ Calcd. for C_20_H_21_N_2_O_5_ 369.1450; Found 369.1443.

*3-(8,11-Oxapentacyclo[5.4.0.0^2,6^.0^3,10^.0^5,9^]undecane-8-amino)propyl 2-nitrobenzoate* (**7**). A brownish yellow and transparent thick liquid, yield 30.67%. FT-IR (ATR): ν_max_ (cm^−1^) = 3329.05, 2963.70, 2863.58, 1729.28, 1608.97, 1578.27. ^1^H-NMR (400 MHz, CDCl_3_): δ 7.90 (dd, *J* = 7.8, 1.4 Hz, 1H), 7.75 (dd, *J* = 7.4, 1.8 Hz, 1H), 7.65 (dtd, *J* = 18.2, 7.6, 1.6 Hz, 2H), 4.61 (t, *J* = 5.2 Hz, 1H), 4.42 (t, *J* = 6.4 Hz, 2H), 2.91 (td, *J* = 7, 2.8 Hz, 2H), 2.83–2.40 (m, 8H), 1.90 (quin, *J* = 6.8 Hz, 2H) 1.71 (ABq, *J* = 10.8 Hz, 2H). ^13^C-NMR (101 MHz, CDCl_3_) and DEPT-135: δ 165.42 (C=O), 148.29 (ArC), 132.83 (ArCH), 131.72 (ArCH), 129.94 (ArCH), 127.70 (ArC), 123.84 (ArCH), 110.25 (C-8), 82.40 (C-11), 64.68 (CH_2_O), 55.14 (CH), 54.72 (CH), 44.83 (CH), 44.64 (CH), 44.50 (CH), 43.26 (CH_2_NH), 43.08 (CH), 41.88 (CH), 41.53 (CH), 40.29 (CH_2_), 29.88 (CH_2_). HRMS (ESI/TOF) *m*/*z*: [M + H]^+^ Calcd. for C_21_H_23_N_2_O_5_ 383.1607; Found 383.1599.

*3-(8,11-Oxapentacyclo[5.4.0.0^2,6^.0^3,10^.0^5,9^]undecane-8-amino)propyl 3-nitrobenzoate* (**8**). A brownish yellow and transparent thick liquid, yield: 48.35%. FT-IR (ATR): ν_max_ (cm^−1^) = 2963.42, 2863.86, 1722.58, 1616.52, 1531.37. ^1^H-NMR (400 MHz, CDCl_3_): δ 8.85 (s, 1H), 8.42 (d, *J* = 9.6 Hz, 1H), 8.36 (d, *J* = 8 Hz, 1H), 7.66 (t, *J* = 8Hz, 1H), 4.62 (t, *J* = 5.2 Hz, 1H), 4.48 (t, *J* = 6.4 Hz, 2H), 2.99 (td, *J* = 7 Hz, *J* = 3.2 Hz, 2H), 2.84–2.41 (m, 8H), 1.99 (quin, *J* = 6.8 Hz, 2H) 1.72 (ABq, *J* = 10.8 Hz, 2H). ^13^C-NMR (101 MHz, CDCl_3_) and DEPT-135: δ 164.50 (C=O), 148.27 (ArC), 135.30 (ArCH), 132.10 (ArC), 129.64 (ArCH), 127.37 (ArCH), 124.54 (ArCH), 110.67 (C-8), 82.40 (C-11), 64.14 (CH_2_O), 55.19 (CH), 54.75 (CH), 44.83 (CH), 44.60 (CH), 44.53 (CH), 43.25 (CH_2_NH), 43.10 (CH), 41.87 (CH), 41.54 (CH), 40.48 (CH_2_), 30.14 (CH_2_). HRMS (ESI/TOF) *m*/*z*: [M + H]^+^ Calcd. for C_21_H_23_N_2_O_5_ 383.1607; Found 383.1604.

*3-(8,11-Oxapentacyclo[5.4.0.0^2,6^.0^3,10^.0^5,9^]undecane-8-amino)propyl 4-nitrobenzoate* (**9**). A creamy white waxy solid, yield: 40.96%, m.p.: 70–75 °C. FT-IR (ATR): ν_max_ (cm^−1^) = 3308.75, 2964.14, 2850.24, 1709.53, 1603.35, 1519.04. ^1^H-NMR (400 MHz, CDCl_3_): δ 8.27 (d, *J* = 8.8 Hz, 2H), 8.20 (d, *J* = 8.8 Hz, 2H), 4.63 (t, *J* = 5.2 Hz, 1H), 4.46 (t, *J* = 6.4 Hz, 2H), 2.98 (td, *J* = 7, 3.6 Hz, 2H), 2.84–2.70 (m, 3H), 2.6 (q, *J* = 6.8 Hz, 2H), 2.53–2.49 (m, 3H), 2.42 (t, *J* = 4.8 Hz, 1NH), 1.98 (quin, *J* = 6.7 Hz, 2H) 1.71 (ABq, *J* = 10.8 Hz, 2H). ^13^C-NMR (101 MHz, CDCl_3_) and DEPT-135: δ 164.71 (C=O), 150.51 (ArC), 135.72 (ArC), 130.70 (ArCH), 123.54 (ArCH), 109.99 (C-8), 82.48 (C-11), 64.16 (CH_2_O), 55.27 (CH), 54.75 (CH), 44.84 (CH), 44.64 (CH), 44.53 (CH), 43.28 (CH_2_NH), 43.07 (CH), 41.89 (CH), 41.54 (CH), 40.45 (CH_2_), 30.13 (CH_2_). HRMS (ESI/TOF) *m*/*z*: [M + H]^+^ Calcd. for C_21_H_23_N_2_O_5_ 383.1607; Found 383.1600.

*3-(8,11-Oxapentacyclo[5.4.0.0^2,6^.0^3,10^.0^5,9^]undecane-8-amino)propyl benzoate* (**10**). A colorless and transparent thick liquid, yield: 30%. FT-IR (ATR): ν_max_ (cm^−1^) = 3329.96, 2963.35, 2863.19, 1715.27, 1601.74, 1584.17. ^1^H-NMR (400 MHz, CDCl_3_): δ 8.03 (d, *J* = 7.2 Hz, 2H), 7.55 (t, *J* = 7.6 Hz, 1H), 7.43 (t, *J* = 7.6 Hz, 2H), 4.62 (t, *J* = 5.2 Hz, 1H), 4.41 (t, *J* = 6.4 Hz, 2H), 2.98 (td, *J* = 7, 3.6 Hz, 2H), 2.83–2.47 (m, 7H), 2.41 (t, *J* = 4.8 Hz, 1H), 1.96 (quin, *J* = 6.6 Hz, 2H) 1.71 (ABq, *J* = 10.8 Hz, 2H). ^13^C-NMR (101 MHz, CDCl_3_) and DEPT-135: δ 166.62 (C=O), 132.86 (ArCH), 130.34 (ArC), 129.56 (ArCH), 128.32 (ArCH), 109.70 (C-8), 82.47 (C-11), 63.17 (CH_2_O), 55.26 (CH), 54.72 (CH), 44.83 (CH), 44.68 (CH), 44.51 (CH), 43.29 (CH_2_NH), 43.06 (CH), 41.90 (CH), 41.54 (CH), 40.53 (CH_2_), 30.29 (CH_2_). HRMS (ESI/TOF) *m*/*z*: [M + H]^+^ Calcd. for C_21_H_24_NO_3_ 338.1756; Found 338.1752.

*2-(8,11-Oxapentacyclo[5.4.0.0^2,6^.0^3,10^.0^5,9^]undecane-8-amino)ethyl benzoate* (**11**). An off-white crystalline solid, yield 36.52%, m.p.: 63–65 °C. FT-IR (ATR): ν_max_ (cm^−1^) = 3315.66, 2949.23, 2868.14, 1709.85, 1602.93, 1584.49. ^1^H-NMR (600 MHz, CDCl_3_): δ 8.04 (d, *J* = 7.2 Hz, 2H), 7.55 (t, *J* = 7.8 Hz, 1H), 7.43 (t, *J* = 7.8 Hz, 2H), 4.64 (t, *J* = 5.4 Hz, 1H), 4.42 (t, *J* = 6 Hz, 2H), 3.23–3.16 (m, 2H), 2.84–2.77 (m, 2H), 2.72 (q, *J* = 6.6 Hz, 1H), 2.60 (q, *J* = 6.6 Hz, 1H), 2.56–2.52 (m, 2H), 2.50 (t, *J* = 4.8 Hz, 1H), 2.42 (t, *J* = 4.8 Hz, 1H), 1.72 (ABq, *J* = 10.8 Hz, 2H). ^13^C-NMR (101 MHz, CDCl_3_) and DEPT-135: δ 166.55 (C=O), 132.92 (ArCH), 130.23 (ArC) 129.63 (ArCH), 128.33 (ArCH), 109.32 (C-8), 82.58 (C-11), 65.64 (CH_2_O), 55.39 (CH), 54.77 (CH), 44.86 (CH), 44.75 (CH), 44.56 (CH), 43.32 (CH_2_NH), 43.08 (CH), 42.59 (CH_2_), 41.94 (CH), 41.55 (CH). HRMS (ESI/TOF) *m*/*z*: [M + H]^+^ Calcd. for C_20_H_22_NO_3_ 324.1600; Found 324.1598.

#### 3.1.5. Procedure for Synthesis of 8-Benzylamino-8,11-oxapentacyclo[5.4.0.0^2,6^.0^3,10^.0^5,9^]undecane (NGP 1-01) (**12**)

The pentacyclo[5.4.1.0^2,6^.0^3,10^.0^5,9^]undecane-8-11-dione (5 g, 28.74 mmol) was dissolved in dry tetrahydrofuran (50 mL) taken in a 250-mL round-bottom flask. The round-bottom flask was kept in an external ice bath with stirring, so as to maintain the temperature at 0 °C. Benzyl amine (3.08 g, 28.74 mmol) was added drop-wise using a dropping funnel, while stirring at low temperature. The carbinolamine started to precipitate in 10 min, and the mixture was stirred for another 20 min, after which dry methanol (30 mL) was added, and the mixture was removed from the ice bath. Once the precipitate dissolved, sodium borohydride (1.5 g) was added slowly, and the mixture was stirred at room temperature overnight. The solvent was evaporated under vacuum, and water was added, followed by extraction with dichloromethane (4 × 50 mL). Anhydrous sodium sulphate was used to dry the combined organic fractions, which on evaporation gave a brownish reaction mixture. The mixture was purified using column chromatography (30% ethyl acetate in hexane), and a pure product was obtained by crystallization from ethyl acetate as white needle-shaped crystals (3.31 g, 12.47 mmol). The physical and spectral data corresponded to that described in the literature [30,31]. A white needle-shaped crystalline solid, yield: 43.44%, m.p.: 85–86 °C. FT-IR (ATR): ν_max_ (cm^−1^) = 3331.55, 3306.15, 2969.82, 2940.67, 2860.54, 1485.70, 1451.27. ^1^H-NMR (400 MHz, CDCl_3_): δ 7.37 (d, *J* = 6.8 Hz, 2H), 7.31 (t, *J* = 7.4 Hz, 2H), 7.23 (t, *J* = 7.0 Hz, 2H), 4.67 (t, *J* = 5.4 Hz, 1H), 4.00 (ABq, *J* = 13.2 Hz, 2H), 2.87–2.79 (m, 2H), 2.73 (appt, *J* = 6.4 Hz, 1H), 2.63–2.53 (m, 4H), 2.43 (t, *J* = 4.8 Hz, 1H), 2.21 (brs, 1H, NH), 1.73 (ABq, *J* = 10.4 Hz, 2H). ^13^C-NMR (101 MHz, CDCl_3_): δ 140.83 (ArC), 128.39 (ArCH), 127.92 (ArCH), 126.97 (ArCH), 109.62 (C-8), 82.53 (C-11), 55.26, 54.80, 47.83 (CH_2_NH), 44.89, 44.59, 43.30, 43.17, 42.03, 41.57. HRMS (ESI/TOF) *m*/*z*: [M + H]^+^ Calcd. for C_18_H_20_NO 266.1545; Found 266.1540.

#### 3.1.6. Procedure for Synthesis of Oxa-Bridged Monoamine Cage **13** and Aza-Bridged Monoamine Cage **21**

##### 8-Amino-8,11-oxapentacyclo[5.4.0.0^2,6^.0^3,10^.0^5,9^]undecane (**13**)

A solution of NGP1-01, **12** (1 g, 3.77 mmol) in ethanol (10 mL) was placed in a pressure reactor along with a stirring bar. The pressure reactor was purged with nitrogen as an inert gas. To this solution was added 0.188 g (calculated as 50 mg per mmol of substrate) of 10% Pd/C. Special care was taken to make sure that the Pd/C was added in small amounts while opening the pressure reactor for as little time as possible, considering the pyrophoric nature of dry Pd/C. After the addition of Pd/C, the pressure tube was sealed properly, and attached to the hydrogen supply. Hydrogen was flushed through the sealed pressure reactor six times before filling it with hydrogen gas at 206 kPa pressure. The mixture was stirred for 14 h, while maintaining the internal temperature of the reaction at 50 °C. Completion of the reaction was confirmed by TLC. On completion of the reaction, the reaction mixture was filtered under vacuum through a sintered glass funnel. Residual Pd/C waste was transferred to a labeled container that contained water. The filtrate was concentrated, and 10 mL of saturated NaHCO_3_ was added. The resulting reaction mixture was extracted with (3 × 7 mL) dichloromethane. Anhydrous sodium sulphate was added to the organic fractions, and it was filtered to get a clear organic layer. The dichloromethane was evaporated under reduced pressure to get a white reaction mixture. A pure mono amine cage product (0.33 g, 1.88 mmol, 49.86%) was obtained by column chromatography using pure ethyl acetate and 10% ethanol in ethyl acetate as mobile phase. A white solid, yield: 49.86%, m.p.: 166–168 °C. FT-IR (ATR): ν_max_ (cm^−1^) = 3387.20, 3314.41, 2964.62, 2860.92, 1623.26, 1451.59, 1371.64. ^1^H-NMR (200 MHz, CDCl_3_): δ 4.43 (t, *J* = 5.2 Hz, 1H), 2.70–2.08 (m, 8H), 1.53 (ABq, *J* = 10.6 Hz, 2H). ^13^C-NMR (75 MHz, CDCl_3_): δ 106.31, 83.05, 57.49, 55.16, 47.00, 44.90, 43.40, 42.44, 41.46. MS (ESI) *m*/*z*: 176.24 (M + H^+^), 177.20, 198.24, 199.23, 214.20, 236.16, 373.30.

##### 4-Azahexacyclo[5.4.1.0^2,6^.0^3,10^.0^5,9^.0^8,11^]dodecan-3-ol (**21**)

The synthesis of compound **21** was carried out from compound **20** by the same procedure as that for the synthesis of compound **13**, except that the hydrogen gas pressure was maintained at 345 kPa. The spectroscopic data correlated with the literature [18].

A white solid, m.p.: 183–186 °C. FT-IR (ATR): ν_max_ (cm^−1^) = 3237.59, 3065.07, 2951.94, 2865.35, 1456.71, 1351.95, 1320.89. ^1^H-NMR (400 MHz, CDCl_3_): δ 3.56 (t, *J* = 5.0 Hz, 1H), 2.85–2.80 (m, 1H), 2.78–2.70 (m, 2H), 2.65–2.61 (m, 2H), 2.50–2.42 (m, 3H), 1.67 (ABq, *J* = 10.4 Hz, 2H). ^13^C-NMR (101 MHz, CDCl_3_): δ 60.89 (d), 55.49 (d), 54.46, 46.13 (d), 45.00, 43.52 (d), 42.56, 42.02 (d). MS (ESI) *m*/*z*: 176.02 (M + H^+^), 177.04.

#### 3.1.7. Procedure for Synthesis of Compounds (**14**–**18**) and (**22**)

##### 8-(2-Nitrobenzylamino)-8,11-oxapentacyclo[5.4.0.0^2,6^.0^3,10^.0^5,9^]undecane (**14**)

Monoamine cage, 8-Amino-8,11-oxapentacyclo[5.4.0.0^2,6^.0^3,10^.0^5,9^]undecane **13** (0.7 g, 3.99 mmol) was dissolved in 40 mL of dry acetonitrile in a round-bottom flask. Potassium carbonate (0.828 g, 5.99 mmol) and tetrabutylammonium hydrogensulfate (0.08 g, 0.24 mmol) were added as the base and phase transfer catalyst, respectively, to the solution in the round-bottom flask, and stirred for 10 min. A drying tube filled with indicator silica was fitted on the neck of the round-bottom flask to prevent the atmospheric moisture from entering the contents of the round-bottom flask. 2-Nitrobenzyl bromide (0.863 g, 3.99 mmol) was added to the stirring suspension, and it was heated on a hot plate that maintained the internal temperature of the reaction mixture at 70 °C. A drying tube was fitted on the top end of a condenser that was attached to the neck of the round-bottom flask throughout the reaction. Progress of the reaction was monitored by TLC. After the completion of the reaction (20 h), the K_2_CO_3_ was filtered off using a sintered glass funnel, and washed with acetonitrile. The filtrate and washings were combined, the solvent was distilled off at reduced pressure, and the crude reaction mixture was column chromatographed on silica using 10% to 30% ethyl acetate in hexane as mobile phase to get a thick liquid as the final product, which solidified to a crystalline yellow solid (0.904 g, 2.91 mmol) on long standing. A yellow solid, yield: 72.96%, m.p.: 89–91 °C. FT-IR (ATR): ν_max_ (cm^−1^) = 3305.90, 2954.93, 2869.17, 1731.88, 1610.49, 1578.06, 1520.84. ^1^H-NMR (400 MHz, CDCl_3_): δ 7.92 (dd, *J* = 8 Hz, 0.8 Hz, 1H), 7.67 (d, *J* = 7.2 Hz, 1H), 7.57 (td, *J* = 7.6 Hz, 1.06 Hz, 1H), 7.39 (td, *J* = 8 Hz, 1.0 Hz, 1H), 4.64 (t, *J* = 5.2 Hz, 1H), 4.28 (ABq, *J* = 15.2 Hz, 2H), 2.85–2.70 (m, 3H), 2.62–2.57 (m, 1H), 2.50–2.48 (m, 3H), 2.42 (t, *J* = 4.8 Hz, 1H), 1.72 (ABq, *J* = 10.4 Hz, 2H). ^13^C-NMR (101 MHz, CDCl_3_) and DEPT-135: δ 149.06 (ArC), 136.53 (ArC), 133.02 (ArCH), 130.85 (ArCH), 127.80 (ArCH), 124.61 (ArCH), 109.43 (C-8), 82.61 (C-11), 55.16 (CH), 54.69 (CH), 45.05 (CH_2_NH), 45.04 (CH), 44.87 (CH), 44.52 (CH), 43.32 (CH_2_), 43.16 (CH), 41.92 (CH), 41.54 (CH). HRMS (ESI/TOF) *m*/*z*: [M + H]^+^ Calcd. for C_18_H_19_N_2_O_3_ 311.1396; Found 311.1394.

Compound **13** was reacted with the appropriate nitro benzyl bromide for the synthesis of compounds **15** and **16**, and with *m*-nitrophenethyl bromide for compound **17** using the same procedure as used for compound **14**. Compound **22** was also synthesized using the similar procedure, but by reacting compound **21** with *p*-nitrobenzyl bromide.

*8-(3-Nitrobenzylamino)-8,11-oxapentacyclo[5.4.0.0^2,6^.0^3,10^.0^5,9^]undecane* (**15**). A light yellow crystalline solid, yield: 79.42%, m.p.: 121–123 °C. FT-IR (ATR): ν_max_ (cm^−1^) = 3321.22, 2967.17, 2860.76, 1582.33, 1521.33, 1441.85. ^1^H-NMR (600 MHz, CDCl_3_): δ 8.28 (s, 1H), 8.09 (dd, *J* = 7.8 Hz, 1.5 Hz, 1H), 7.68 (d, *J* = 7.8 Hz, 1H), 7.48 (t, *J* = 7.8 Hz, 1H), 4.67 (t, *J* = 5.1 Hz, 1H), 4.11 (ABq, *J* = 15 Hz, 2H), 2.86–2.83 (m, 1H), 2.82–2.78 (m, 1H), 2.77–2.74 (m, 1H), 2.64–2.60 (m, 1H), 2.56 (t, *J* = 4.8 Hz, 1H), 2.53–2.50 (m, 2H), 2.44 (t, *J* = 4.8 Hz, 1H), 2.35 (brs, 1H, NH), 1.73 (ABq, *J* = 10.2 Hz, 2H). ^13^C-NMR (101 MHz, CDCl_3_) and DEPT-135: δ 148.46 (ArC), 143.51 (ArC), 133.77 (ArCH), 129.17 (ArCH), 122.54 (ArCH), 121.91 (ArCH), 109.42 (C-8), 82.63 (C-11), 55.37 (CH), 54.81 (CH), 46.94 (CH_2_NH), 45.00 (CH), 44.89 (CH), 44.63 (CH), 43.34 (CH_2_), 43.18 (CH), 42.00 (CH), 41.58 (CH). HRMS (ESI/TOF) *m*/*z*: [M + H]^+^ Calcd. for C_18_H_19_N_2_O_3_ 311.1396; Found 311.1395.

*8-(4-Nitrobenzylamino)-8,11-oxapentacyclo[5.4.0.0^2,6^.0^3,10^.0^5,9^]undecane* (**16**). A light yellow solid, yield: 84.34%, m.p.: 144–148 °C. FT-IR (ATR): ν_max_ (cm^−1^) = 3333.26, 2964.21, 2861.80, 1598.72, 1506.38, 1431.06. ^1^H-NMR (400 MHz, CDCl_3_): δ 8.19–8.10 (m, 2H), 7.55–7.44 (m, 2H), 4.67 (t, *J* = 5.2 Hz, 1H), 4.11 (ABq, *J* = 15.2 Hz, 2H), 2.87–2.72 (m, 3H), 2.64–2.60 (m, 1H), 2.53–2.48 (m, 3H), 2.44 (t, *J* = 4.8 Hz, 1H), 2.36 (brs, 1H, NH), 1.73 (ABq, *J* = 10.8 Hz, 2H). ^13^C-NMR (101 MHz, CDCl_3_) and DEPT-135: δ 149.06 (ArC), 146.95 (ArC), 128.23 (ArCH), 123.59 (ArCH), 109.45 (C-8), 82.62 (C-11), 55.38 (CH), 54.79 (CH), 47.14 (CH_2_NH), 44.96 (CH), 44.87 (CH), 44.62 (CH), 43.32 (CH_2_), 43.18 (CH), 41.98 (CH), 41.58 (CH). HRMS (ESI/TOF) *m*/*z*: [M + H]^+^ Calcd. for C_18_H_19_N_2_O_3_ 311.1396; Found 311.1395.

*8-(3-Nitrophenethylamino)-8,11-oxapentacyclo[5.4.0.0^2,6^.0^3,10^.0^5,9^]undecane* (**17**). A thick yellow oil, yield: 11.05%. FT-IR (ATR): ν_max_ (cm^−1^) = 3319.88, 2963.69, 2863.71, 1732.15, 1523.99, 1478.00. ^1^H-NMR (600 MHz, CDCl_3_): δ 8.11 (s, 1H), 8.07 (dd, *J* = 8.4 Hz, 1.2 Hz, 1H), 7.56 (d, *J* = 7.2 Hz, 1H), 7.45 (t, *J* = 7.8 Hz, 1H), 4.62 (t, *J* = 5.1 Hz, 1H), 3.16–3.07 (m, 2H), 2.91 (t, *J* = 7.2 Hz, 2H), 2.82–2.75 (m, 2H), 2.70 (q, *J* = 6.6 Hz, 1H), 2.59 (q, *J* = 6.6 Hz, 1H), 2.50–2.45 (m, 3H), 2.41 (t, *J* = 4.8 Hz, 1H), 1.71 (ABq, *J* = 10.8 Hz, 2H). ^13^C-NMR (101 MHz, CDCl_3_): δ 148.38 (ArC), 142.13 (ArC), 135.08 (ArCH), 129.22 (ArCH), 123.72 (ArCH), 121.35 (ArCH), 82.52 (C-11), 62.96 (C-8), 55.42 (CH), 54.75 (CH), 44.84 (CH_2_NH), 44.63 (CH), 44.58 (CH), 44.53 (CH), 43.31 (CH_2_), 43.07 (CH), 41.91 (CH), 41.55 (CH), 37.04 (Ar-CH_2_). HRMS (ESI/TOF) *m*/*z*: [M + H]^+^ Calcd. for C_19_H_21_N_2_O_3_ 325.1552; Found 325.1550.

*4-(4-Nitrobenzyl)-4-azahexacyclo[5.4.1.0^2,6^.0^3,10^.0^5,9^.0^8,11^]dodecan-3-ol* (**22**). A light yellow solid, yield: 59.53%, m.p.: 165–167 °C. FT-IR (ATR): ν_max_ (cm^−1^) = 2960.45, 2873.53, 1598.38, 1515.06, 1491.83. ^1^H-NMR (400 MHz, CDCl_3_): δ 8.18 (d, *J* = 8.8 Hz, 2H), 7.53 (d, *J* = 8.8 Hz, 2H), 3.91 (d, *J* = 14.8 Hz, 1H, NCH_2_), 3.56 (brs, 1H, NCH_2_), 3.30 (t, *J* = 4.8Hz, 1H, C-11), 2.95 (q, *J* = 6.5 Hz, 1H), 2.81–2.74 (m, 2H), 2.67–2.59 (m, 2H), 2.54–2.51 (m, 2H), 2.44 (t, *J* = 4.8 Hz, 1H), 1.65 (ABq, *J* = 10.6 Hz, 2H). ^13^C-NMR (101 MHz, CDCl_3_): δ 147.31, 146.99, 129.06, 123.60, 65.18, 59.16, 54.96, 51.16, 50.96, 45.59, 44.61, 42.97, 42.31, 41.74, 41.31, 24.23. HRMS (ESI/TOF) *m*/*z*: [M + H]^+^ Calcd. for C_18_H_19_N_2_O_3_ 311.1396; Found 311.1396.

*8-Phenethylamino-8,11-oxapentacyclo[5.4.0.0^2,6^.0^3,10^.0^5,9^]undecane* (**18**). The synthesis of compound **18** was carried out by reacting compound **13** with phenethyl bromide using a similar procedure as used for compounds (**14**–**17**) and **27**, but using the microwave conditions (Power = 250 W, T = 150 °C, P = 200 Psi). The reaction completed after a total reaction time of 3 h (TLC). A whitish thick liquid, yield: 15.41%. FT-IR (ATR): ν_max_ (cm^−1^) = 3322.51, 2962.94, 2862.52, 1732.87, 1603.36, 1496.31, 1453.93. ^1^H-NMR (400 MHz, CDCl_3_): δ 7.30–7.17 (m, 5H), 4.62 (t, *J* = 5.2 Hz, 1H), 3.13–3.02 (m, 2H), 2.81 (t, *J* = 7.4 Hz, 2H), 2.74–2.56 (m, 4H), 2.51–2.48 (m, 3H), 2.41 (t, *J* = 4.8, 1H), 1.71 (ABq, *J* = 10.4, 2H), 1.25 (brs, 1H, NH). ^13^C-NMR (101 MHz, CDCl_3_): δ 139.89 (ArC), 128.75 (ArCH), 128.41 (ArCH), 126.12 (ArCH), 82.45 (C-11), 55.35 (CH), 54.76 (CH), 45.01 (CH_2_), 44.83 (CH), 44.57 (CH), 44.51 (CH), 43.30 (CH_2_NH), 43.07 (CH), 41.94 (CH), 41.54 (CH), 37.24 (Ar-CH_2_). HRMS (ESI/TOF) *m*/*z*: [M + H]^+^ Calcd. for C_19_H_22_NO 280.1701; Found 280.1692.

#### 3.1.8. Procedure for Synthesis of 3-Hydroxy-5-cyano-4-benzyl-4-azahexacyclo[5.4.1.0^2,6^.0^3,10^.0^5,9^.0^8,11^] Dodecane (**23**)

The pentacyclo[5.4.1.0^2,6^.0^3,10^.0^5,9^]undecane-8-11-dione (5 g, 28.74 mmol) was dissolved in dry tetrahydrofuran (50 mL) in a 500-mL round-bottom flask. The round-bottom flask was kept in an external ice bath with stirring, so as to maintain the temperature below 3 °C. Benzylamine (3.08 g, 28.74 mmol) was added drop-wise using a dropping funnel, while stirring at a lower temperature. The carbinolamine started to precipitate in 10 min, and the mixture was stirred for another 20 min, after which dry methanol (250 mL) and acetic acid (15 mL) were added, and the mixture was removed from the ice bath. Once all of the precipitate had dissolved, sodium cyanide (2.11 g, 43.06 mmol) was added portion-wise, and the reaction mixture was stirred overnight. The reaction mixture was concentrated in vacuo, and water (100 mL) was added to the concentrated reaction mixture in the round-bottom flask. Solid NaHCO_3_ was added to the resulting suspension until the effervescence resulting from carbon dioxide evolution ceased. Additional solid NaHCO_3_ was added to the suspension, and it was extracted with dichloromethane (4 × 50 mL). The combined organic fraction was dried with anhydrous sodium sulphate, and the dichloromethane was evaporated under reduced pressure. The solid reaction mixture obtained was crystallized from isopropanol to give a white solid, which was further recrystallized from dichloromethane to give white long needle-shaped crystals of the product, yield: 39.61%, m.p.: 152–155 °C. FT-IR (ATR): ν_max_ (cm^−1^) = 3093.63, 2957.91, 2871.24, 2237.59, 1606.05, 1496.74, 1454.51, 1371.09, 1331.92. ^1^H-NMR (200 MHz, CDCl_3_): δ 7.29–7.13 (m, 5H), 3.67 (s, 2H, NCH_2_), 3.15–2.40 (m, 7H), 1.96 (brs, 1H, OH), 1.63 (ABq, *J* = 11.0 Hz, 2H). ^13^C-NMR (75 MHz, CDCl_3_): δ 138.70, 128.67, 128.33, 127.26, 119.92, 101.47, 57.18, 53.67, 51.08, 48.07, 45.76, 44.47, 42.50, 42.23, 40.53, 40.18. HRMS (ESI/TOF) *m*/*z*: [M + H]^+^ Calcd. for C_19_H_19_N_2_O 291.1497; Found 291.1488.

### 3.2. Biological Studies

#### 3.2.1. Cytotoxicity Studies

PC12 cells were seeded into 96-well microtiter plates at a cell density of 60,000 cells/mL, using 100 μL of the cell suspension per well (6000 cells/well). The microtiter plates were incubated for 24 h in a humidified incubator and 5% CO_2_ at 37 °C, prior to addition of the compounds. The compounds were diluted to double the desired final maximum test concentration with a complete medium. Working concentrations were prepared by serial dilutions ranging between 3.125–400 µM. Aliquots of 100 µL of these different compound dilutions were added to the appropriate microtiter wells already containing 100 µL of medium, resulting in the required final compound concentrations (ranging between 1.5265–200 µM). Following test sample addition, plates were incubated for 48 h at 37 °C and 5% CO_2_ in a humidified incubator.

After 48 h, the treatments were removed via aspiration, 100 μL of MTT (0.5 mg/mL) [20] was dissolved in complete medium and added to each well, and the treatments were incubated for 3 h at 37 °C. After 3 h, MTT was removed via aspiration, 100 μL of DMSO was added to each well to dissolve the formazan crystals, and the absorbance was read at 540 nm using a BioTek^®^ PowerWave XS spectrophotometer. Cell viability was determined using four replicate wells for each concentration. Untreated cells were considered to have 100% cell viability (i.e., the mean optical density (OD) of the untreated wells = 100% viability). Cell viability in test wells was calculated relative to the untreated control, and expressed as a percentage.

#### 3.2.2. Neuroprotection Studies

PC12 cells were seeded into 96-well microtiter plates at a cell density of 100,000 cells/mL, using 100 μL of the cell suspension per well (10,000 cells/well). The microtiter plates were incubated for 24 h in a humidified incubator and 5% CO_2_ at 37 °C, prior to the addition of the compounds. The compounds were diluted to triple the desired working test concentrations (i.e., 15 µM, 75 µM, and 150 µM) with complete medium [containing RPMI, 10% foetal bovine serum, 5% horse serum, and 1% penicillin/streptomycin (Biowest, Nuaille, France)]. Aliquots (50 µL) of the working concentrations were added to the appropriate microtiter wells, yielding test concentrations of 5 µM, 25 µM, and 50 µM of each compound. *N*-acetyl-l-cysteine (Sigma) was used as the positive control. An *N*-acetyl-l-cysteine stock (500 mM) was prepared in sterile water and filter-sterilized. Aliquots (50 µL) of the working *N*-acetyl-l-cysteine concentration (1.5 mM) were added to the appropriate microtiter wells, yielding a final test concentration of 500 µM. Microtiter plates were incubated for 24 h in a humidified incubator and 5% CO_2_ at 37 °C, following test sample addition. Paraquat dichloride (Sigma, St. Louis, MO, USA) was used as a neurotoxin. A paraquat dichloride stock (10 mM) was prepared in sterile water and sterilized by filtration prior to use. Aliquots (50 µL) of a working paraquat dichloride solution (1 mM) were added to half of the microtiter plates’ wells, yielding a test concentration of 250 µM. The addition of 50 µL of complete medium to the remaining wells served as sample controls representing cell viability in the absence of added neurotoxin, but in the presence of the test compound. Microtiter plates were further incubated for 24 h in a humidified incubator and 5% CO_2_ at 37 °C, following neurotoxin addition.

After 20 h of neurotoxin treatment, 20 μL of CellTiter-Blue^®^ Reagent (Promega, Madison, MO, USA) was added to each well, and it was incubated for a further 4 h in a humidified incubator and 5% CO_2_ at 37 °C. Fluorescence was measured using a BioTek SYNERGY Mx fluorometer (Winooski, VT, USA) at excitation and emission wavelengths of 560 nm and 590 nm, respectively. The cell viability was determined using three replicate wells for each treatment, and percentage protection was calculated as the difference between paraquat-containing and paraquat-free wells.

#### 3.2.3. NMDA-Mediated and Voltage-Gated Ca^2+^ Influx Studies

The study protocol was approved by the institutional ethical committee (ethics approval number: SRIRC2012/06/13) for research, and university guidelines were followed throughout the experiments. Adult Wistar rats (250–300 g) were used. They were allowed free access to food and water, and maintained at a 12 h day/night cycle.

##### Preparation of Synaptoneurosomes

Synaptoneurosomes were prepared by a method validated for synaptosomal preparation [25]. Freshly isolated rat brain was put into 15 mL of ice-cooled incubation buffer (118 mM NaCl, 4.7 mM KCl, 0.1 mM CaCl_2_·2H_2_O, 20 mM HEPES and 30.9 mM glucose monohydrate) taken in a 50-mL Falcon tube. Incubation buffer was decanted into the waste, and 15 mL of incubation buffer was added into the falcon tube holding the brain. The incubation buffer was again decanted to get the clear brain. Then, 20 mL of ice-cooled incubation buffer was transferred to the Falcon tube with the brain. The contents of the Falcon tube were transferred into the glass homogenizer. The brain tissue was homogenized by eight strokes of 3 s each. The tissue suspension was transferred into two 15-mL Falcon tubes, so that each one had approximately 10 mL of the tissue suspension. The tubes were then centrifuged at 1100× *g* for 5 min at 4 °C. After the centrifugation, the supernatant of both of the tubes was transferred to another 50-mL Falcon tube, and kept in an ice-box. This supernatant was then divided as 2-mL aliquots in pre-weighed 2-mL Eppendorf microfuge tubes. The microfuge tubes were centrifuged at 15,000× *g* for 20 min at 4 °C. The supernatant in these tubes was discarded, and the tubes were again weighed to find the weight of the synaptoneurosomes/protein pellet in each Eppendorf tube. The pellets were further diluted with calcium-free buffer (with the same composition as the incubation buffer, but without CaCl_2_·2H_2_O) to get a protein concentration of 3 mg/mL. The resulting suspensions were vortexed to thoroughly mix the suspension. This suspension (1990 µL) was added into another 2-mL Eppendorf tube, and allowed to reach room temperature, followed by a 10-µL addition of 1 mM of solution of Fura-2/AM in DMSO, making sure that all of the solutions involving Fura-2/AM were protected from light at all times. The solution was gently vortexed to mix the Fura-2/AM thoroughly, and then incubated at 37 °C for 30 min, with shaking at the rate of 280 rpm. The suspension was again centrifuged at 15,000× *g* for 5 min to remove the extra-synaptoneurosomal Fura-2/AM. The supernantant was decanted, and the resulting pellets were re-suspended in calcium-containing buffer (with the same composition as the incubation buffer, but 2 mM CaCl_2_·2H_2_O). The re-suspended suspension was vortexed to make it uniform. This solution, which had a synaptoneurosome/protein concentration of 3 mg/mL, was further diluted with calcium-containing buffer so that the final concentration of the suspension was 0.6 mg/mL. This suspension was kept in the dark at room temperature for further use in the next step of the experiment.

##### Measurement of Intracellular Calcium for NMDA-Mediated Studies

Stock solutions of all of the test compounds (10 mM) were prepared by dissolving them in DMSO. Stock solutions (2 µL) of each of the reference and the test compounds were transferred into individual wells on a 96-well plate in triplicate. The stock solutions in the wells were further diluted with 200 µL of synaptoneurosomal-Fura-2/AM solution, so that the final concentration of the reference and the test compounds in the wells was 100 µM. The well plate was then placed into the fluorescent plate reader, where incubation was done at 37 °C for 30 min, followed by shaking for 10 s before fluorescent readings. Dual wavelength excitation was done at 340 nm and 380 nm, whereas emission fluorescence was measured at 510 nm. The fluorescent measurements of each well were done at intervals of 0.5 s for 35 s. Then, 10 µL of stimulation buffer (0.1 mM CaCl_2_·2H_2_O, 0.1 mM glycine, and 0.1 mM NMDA) was introduced in the wells at 10 s after initiation of the experiment by an auto-injector built into the plate reader to activate NMDAR-mediated calcium influx. The experiments were repeated three times on different tissue preparations.

##### Measurement of Intracellular Calcium for Voltage-Gated Studies

The same procedure was followed as for the NMDA-mediated Ca^2+^ influx studies, except that the stimulation buffer was replaced by a depolarizing buffer (5.4 mM NaCl, 140 mM KCl, 1.4 mM CaCl_2_·2H_2_O, 20 mM HEPES, 5.5 mM glucose monohydrate, 10 mM NaHCO_3_, 0.6 mM KH_2_PO_4_, 0.6 mM Na_2_HPO_4_.12H_2_O and 0.9 mM MgSO_4_).

##### Calculation of Percentage Inhibition in NMDA and VG-Mediated Ca^2+^ Influx

The fluorescent data of each cell was expressed as the ratio of fluorescence intensity at 340 nm and 380 nm. The concentration of free intracellular Ca^2+^ was proportional to ratio of fluorescence at 340/380 nm. The average ratio before stimulation was subtracted from the average of the ratio over 25 s after stimulation to get the net Ca^2+^ influx. This calculation was performed for all of the individual wells, and the mean was calculated for all of the wells with the same test compound to give a mean Ca^2+^ influx as [*R*]. These [Ca^2+^] values were used to calculate the percentage inhibition of the test compounds relative to the control by using the following formula:

$$\%\;\mathrm{Inhibition}=\;\frac{\left[R\right]control\;-\left[R\right]test}{\left[R\right]control}\;\times 100$$

[*R*]control = Mean Ca^2+^ influx of control
[*R*]test = Mean Ca^2+^ influx of test compound

#### 3.2.4. *S*-Nitrosylation Studies

Equimolar quantities of cysteine–glycine and a NO donor were taken in Eppendorf microfuge tubes. This was achieved by mixing 200 µL of 4 mM Cys–glyc solution, and 80 µL of 10 mM NO-donor stock solution in 2-mL Eppendorf microfuge tubes. The mixture was stirred at room temperature in the dark for one hour; then, 200 µL of 20 mM methyl methanethiosulfonate (MMTS) solution was added to each microfuge tube, which were incubated at 50 °C for 20 min with frequent shaking. To each microfuge tube, 200 µL of acetone was added, and they were vortexed for 10 min. No MMTS precipitates were formed, contrary to the reported methods. An aliquot of 85 µL was transferred from each Eppendorf microfuge tube to a separate well in the microplate, and 25 µL of biotin-HPDP (4 mM) was added to each well in the microplate. The UV absorbance at 343 nm was measured for up to 15 min, with five-minute time intervals. The absorbance of each sample test run was zeroed, with the absorbance of its reagent blank solution. For a reagent blank sample, the same procedure was used, but without the addition of the cys–gly dipeptide solution, as this was the only solution in the assay without any UV absorbance ability. For the control, the same procedure was followed, but without the addition of NO-donor compounds and MMTS, allowing the cysteine to react completely with the biotin-HPDP and give an indicative UV absorbance for 100% *S*-nitrosylation. The absorbance of the control solution was also adjusted with the absorbance of its own blank solution, which had only HEN buffer and biotin-HPDP.

## 4. Conclusions

As is evident from the results obtained in the five different biological studies (Table 3) for the tested compounds, it was difficult to draw a simple correlation that could explain the results across all of the biological study domains. The three compounds (**8**, **4**, and **6**) with the best neuroprotective behavior also had a high to medium inhibitory effect on NMDA-mediated and voltage-mediated calcium influx. However, not all of the compounds with potent channel inhibitory activity showed good neuroprotection, e.g., compound **15** and **9** had the highest NMDAR-inhibiting activity (94.48% and 80.29%, respectively) amongst the tested compounds, but displayed a mediocre neuroprotection behavior (11.21% and 3.42% respectively). This might be because the Ca^2+^ influx inhibitory studies and neuroprotection studies were done on two different biological systems, viz. synaptoneurosomes and PC12 cells, respectively, and evaluated different mechanisms of possible neuroprotection. The compounds with nitro groups generally showed improved NMDA and voltage-mediated calcium influx inhibition and neuroprotection, but the extent of *S*-nitrosylation did not correlate with the neuroprotective and calcium channel activities.

These results reiterate the fact that neuroprotection is a result of multifactorial biochemical mechanisms and interactions. As evident from these compounds, the significant inhibition of calcium flux (NMDA and voltage mediated), and thus possible protection against excitotoxicity, does not necessarily correlate to the neuroprotection observed in, for example, the paraquat assay, which is based on oxidative stress and mitochondrial toxicity. Structures exhibiting activity in diverse neurodegenerative processes and assays could thus be advantageous in terms of multipotent neuroprotection. The channel activity and the NMDAR antagonism is also controlled by the non-covalent interactions between the receptor and the antagonist; this is where the geometric and steric factors sometimes can dominate the other biochemical factors, as has been suggested in earlier studies evaluating the calcium channel activity and the NMDAR antagonism of these structures [27,32].

The absence of any direct correlation between the extent of the nitrosylating ability of these cage amines and the inhibition of calcium influx is in agreement with a previous report [33]. For the compounds not showing the expected neuroprotection or calcium channel inhibition in spite of some appreciable *S*-nitrosylation, the extent of nitrosylation, and hence the modulation of the calcium channel, might not be sufficient to negate the other factors contributing towards the inhibition of calcium influx. This hypothesis can be established by attaching NO-donating groups with higher *S*-nitrosylating abilities to these compounds, and testing their effect on NMDA and depolarization-induced calcium influx.

One interpretation of these biological data is that the presence of a nitro group on the phenyl ring in general enhanced the neuroprotective and calcium channel activities. Apart from affecting the *S*-nitrosylation, the nitro group also affects the π-electron density on the phenyl ring by its electron withdrawing effect. One suggested hypothesis for the functional antagonism of such cage compounds was that the phenyl ring undergoes π–π interactions with aromatic amino acids located at the entrance of the NMDA receptor [27]. This helps the molecule become anchored by the phenyl ring, so that the cage enters through the length of the channel, and interacts at its binding site. The electron withdrawing effect of the nitro group may provide a favorable electron density for effective π–π interactions. According to a mathematical model based on σ and π atomic charges, relative orientations, and van der Waals interactions to determine the electrostatics of the substituent effect [34], electron withdrawing groups decrease the negative quadropole of the aromatic ring, and thus favour the parallel displaced and sandwich conformations. This could explain the better calcium channel inhibitory properties of compounds with nitro groups, by better anchoring of these molecules at the entrance of the calcium channel.

The above studies reveal important aspects of the structure–activity relationships of pentacycloundecylamine neuroprotective compounds across different biological study realms. The observed neuroprotective, NMDAR, and VGCC channel inhibition and nitrosylating abilities of NO-donating compounds, combined with the very good cytotoxicity profiles of all of the compounds, provide a useful contribution to multitarget neuroprotective drug discovery.

The selected approach in the design of the compounds could be translated to designing compounds with different chemical functionalities and for different therapeutic targets. This has paved the way for further research, which is not limited to, but may include using better NO donors on the same/similar molecular scaffolds, incorporating similar aspects of *S*-nitrosylation for other therapeutic compounds, and further in vivo studies of these compounds that could shed light on the functional application of these compounds.

## Figures and Tables

**Table 1:** Cytotoxicity profile of the test compounds as % viability from MTT assay.

Code	% Viability at Different Concentrations							
	200 µM	100 µM	50 µM	25 µM	12.5 µM	6.25 µM	3.13 µM	1.56 µM
**4**	84.55	97.24	93.68	95.46	96.02	98.67	98.85	95.85
**5**	85.90	91.47	84.30	92.58	100.18	90.40	95.10	98.68
**6**	75.47	98.77	98.27	103.91	99.17	97.12	94.12	106.21
**7**	102.42	105.10	109.14	116.15	110.57	117.52	106.64	110.51
**8**	96.43	108.25	111.05	112.36	109.39	114.19	111.45	116.13
**9**	121.08	122.17	113.29	111.37	109.14	113.08	113.76	115.58
**10**	117.90	120.65	117.10	112.69	116.29	111.34	116.34	117.53
**11**	98.53	101.52	97.62	104.37	101.06	104.19	103.87	101.56
**12**	90.55	103.71	100.13	109.19	98.65	86.40	92.88	99.39
**14**	68.33	108.98	90.37	98.13	100.56	96.09	109.83	104.97
**15**	79.46	93.89	109.08	115.43	102.87	105.63	101.42	107.42
**16**	81.07	85.38	93.21	100.70	96.06	106.68	107.63	109.98
**17**	61.16	102.19	102.91	107.85	97.14	103.35	102.25	110.06
**18**	96.82	94.19	92.45	105.69	94.99	90.97	95.88	102.41
**20**	63.05	80.70	88.05	90.08	90.57	93.91	90.68	99.67
**22**	72.98	112.89	130.70	128.28	118.47	94.80	105.84	115.89
**23**	127.86	126.76	128.73	123.01	132.12	102.78	110.27	99.59

**Table 2:** Percentage of the neuroprotection results of the test compounds at three different concentrations.

Code	%Neuroprotection 5 µM	%Neuroprotection 25 µM	%Neuroprotection 50 µM
**4**	−8.94	21.08	28.17 *
**5**	−13.47	24.48	−23.05
**6**	6.15	−0.66	26.55 *
**7**	14.53	5.98	−1.59
**8**	−10.90	9.95	39.62 *
**9**	−9.58	−7.78	3.42
**10**	−12.54	−3.92	9.52
**11**	14.25	9.37	1.38
**12**	2.88	19.88 *	3.38
**14**	8.04	21.55	24.87
**15**	4.40	4.68	11.21
**16**	6.02	9.68 *	18.71 **
**17**	5.95	5.72	16.69
**18**	12.72	5.52	−8.01
**20**	−1.58	−15.46	0.29
**22**	−5.36	−6.19	24.92 *
**23**	6.40	17.49*	15.33 *

The asterisks indicate the statistical significance (*, *p* < 0.05 and **, *p* < 0.005).

**Table 3:** Consolidated biological results of the test compounds.

Code	%CYT 50 µM	%NP 50 µM	%NMDA 100 µM	%VG 100 µM	%S-NO
**4**	93.68	28.17 *	51.04	30.54	8.14 *
**5**	84.3	24.48 at 25 μM	57.74 *	37.91	7.81 *
**6**	98.27	26.55 *	62.17 ***	39.95 *	7.76 *
**7**	109.14	14.53 at 5 μM	49.26 *	38.51	1.61
**8**	111.05	39.62 *	71.66 ***	41.18 *	2.09
**9**	113.29	3.42	80.29 ***	23.61	2.42
**10**	117.1	9.52	62.92 **	46.03 *	n. d.
**11**	97.62	14.25 at 5 μM	41.88	30.33	n. d.
**12**	100.13	19.88 * at 25 μM	50.84 **	30.71	n. d.
**14**	90.37	24.87	53.19 *	36.79	0.68
**15**	109.08	11.21	94.48 ****	29.35	7.89 *
**16**	93.21	18.71 **	59.6*	47.15 **	9.77 **
**17**	102.91	16.69	50.47	38.91 *	8.34 *
**18**	92.45	12.72 at 5 μM	55.43 *	49.69 **	n. d.
**20**	88.05	0.29	24.92	35.69	n. d.
**22**	130.70	24.92 *	55.22 *	43.76 *	5.40
**23**	128.73	15.33 *	30.78	26.68	n. d.

CYT = % viability at 50 μM concentration, NP = % neuroprotection of test compounds when tested against neurotoxin paraquat at 50-μM concentration, unless specified in the table, NMDA = Mean percentage of inhibition of NMDA-mediated calcium influx when tested with murine synaptoneurosomes at 100-μM concentration of test compounds, VG = Mean percentage of inhibition of voltage-gated calcium influx when tested with murine synaptoneurosome at 100-μM concentration of test compounds, S-NO = Percent of S-nitrosylation caused by the test compounds relative to the control. n.d. stands for not determined. The asterisks indicate the statistical significance (*, *p* < 0.05; **, *p* < 0.005; ***, *p* < 0.001 and ****, *p* < 0.0001) when compared to the control.
